# A comprehensive review of demand side management in distributed grids based on real estate perspectives

**DOI:** 10.1007/s11356-023-25146-x

**Published:** 2023-01-18

**Authors:** Ahmed Tijjani Dahiru, Dzurllkanian Daud, Chee Wei Tan, Zainab Toyin Jagun, Salfarina Samsudin, Abdulhakeem Mohammed Dobi

**Affiliations:** 1 Division of Electrical Power, Faculty of Electrical Engineering, Universiti Teknologi Malaysia, 81310 Skudai, Johor Malaysia; 2 Department of Electrical/Electronics Technology, FCE (Technical), Bichi, Kano State Nigeria; 3 Department of Real Estate, Faculty of Built Environment and Surveying, Universiti Teknologi Malaysia, 81310 Skudai, Johor Malaysia; 4 School of Built Environment Engineering and Computing, Leeds Beckett University, Leeds, LS1 3HE UK; 5 Department of Electrical Engineering, Directorate of Engineering Programmes, Waziri Umaru Federal Polytechnic, Birnin Kebbi, Kebbi State Nigeria

**Keywords:** Renewable energy, Energy management, Distributed grid, Demand side management, Real estate, Building energy management system

## Abstract

A major challenge in renewable energy planning and integration with existing systems is the management of intermittence of the resources and customer demand uncertainties that are attributed to climates. In emerging distributed grids, state-of-the-art optimization techniques were used for cost and reliability objectives. In the existing literature, power dispatch and demand side management schemes were implemented for various techno-economic objectives. In renewable energy-based distributed grids, power dispatch is strategic to system operations. However, demand side management is preferred, as it allows more options for customer participation and active management of energy in buildings. Moreover, the demand side management can simply follow supplies. This paper investigates the implications of demand side management as it affects planning and operations in renewable energy-based distributed grids. Integration of demand side management in customer-oriented plans such as the time-of-use and real-time-pricing on residential and commercial demands is conceptualised to ensure effective customer participation which maintains the valued comforts. Moreover, the optimised tariff integrated demand side management implementations based on the utility-initiated demand response programmes are envisaged to offset conflicting objectives of the economy and customer comforts within residential and commercial demands and are also viewed as a step towards efficient management of energy in buildings.

## Introduction

### Background information

**Fig. 1:**
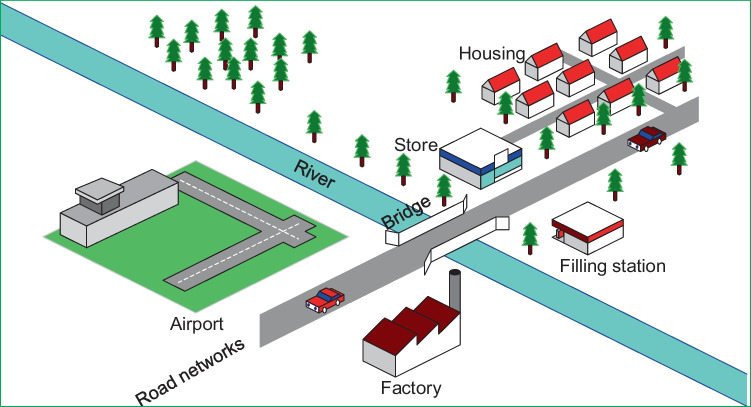
Examples of components in a real estate

**Fig. 2:**
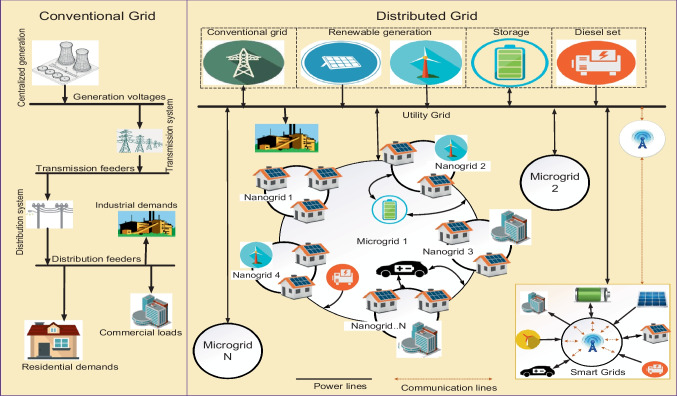
Structural distinctions between conventional grids and distributed generation

An oversized system can be reliable, but costs so much that renders a project uneconomical. Likewise, an undersized system may be economical but tends to be vulnerable to overstretching demands that lead to early failures. Hence, there exist conflicting objectives in-between reliability and economy to be balanced. The main objectives in renewable energy (RE)-based distributed systems are reliability, energy costs, supply availability and emission control. Balancing the foregoing techno-economic objectives in distributed grid planning and operations require defined energy management (EM) schemes at both sides of the metre (the system’s front-of-the-metre and behind-the-metre). The foregoing are issues with EM schemes applied to the supply side (power management) and demand side (load management) in the emerging utilities, the distributed grids (DGs). Demand side management (DSM) is a systematic management of customer demands based on the supply availability (power dispatch) applicable to buildings and, by extension, the general real estate illustrated in Fig. 1. Power dispatch in conventional grids (CGs) is strategic to system operations. However, power dispatch in DGs may be more strategic due to the impacts of uncontrollable RE resources and diversified technologies involved, as illustrated in Fig. 2. Factors of climate change affect both the RE resources used for power generation and control in DG utilities and the customer demands such as the seasonal needs for lighting and space comforts. Thus, reliability challenges are expected in DGs such that what may matter most to the system operations is the optimal implementation of demand side management (DSM). The DSM is known to be flexible enough to be made to follow supplies in the events of intermittent power dispatch and enables customer participation.

The implications of DSM are primarily attributed to the customer side of the electric power distribution networks (PDN). The customer side of the PDN simply comprises electricity demands from the industrial, commercial and residential settings within buildings as components of the real estate establishments. Furthermore, real estate comprises natural or man-made structures that are permanently tied to the land, which includes rivers, vegetation and mineral deposits. Man-made structures can be buildings, roads and bridges. However, the real estate focus of this study constitutes mostly of buildings, which are known to largely contribute to global electricity consumption. In contrast to intangible assets like stocks and bonds, real estate consists of physical land, buildings and other structures. The scope of the rights attached to real property (i.e. the right to enjoy, occupy, use and transfer) is governed by the legal and political procedures that have jurisdiction over the property (Friedman et al. 2017). From a scholarly point of view, the real estate field is an overarching discipline that includes subfields specialising in different aspects of the property development process (Jagun 2020). To be clear, residential real estate refers to either single-family homes or multi-family complexes, depending on the context (Cetin and Novoselac 2015). The most common sort of estate is an area where most people have some level of experience. Residential encompasses many building types, including single-family homes, multi-family complexes, condominiums, townhouses and other housing types.

In modern electricity DGs, buildings play a significant role in shaping city electricity generation, distribution, retails, and utilisation, known as *prosumption* (Dahiru 2021). The role of buildings in the control of greenhouse emissions is evident (Hu et al. 2020). Moreover, electricity is the most commonly used energy source in buildings, and the demands keep increasing. Hence, offsetting the implications of rising demands requires dynamic building energy efficiency strategies at the front-of-the-metre, otherwise referred to as load management or DSM. Building energy management systems (BEMS) enable estate managers and owners to increase energy efficiency in buildings for a reduction in the use of energy. Depending on the nature of the building, several management solutions are utilised for energy savings through the DSM schemes (Mariano-Hernández et al. 2021). There is a need for a BEMS to be designed to respond to the electricity grid conditions for improved efficiency and sustainable energy consumption in buildings (Al Dakheel et al. 2020). The inelasticity in building electricity consumption and stochastic human behaviour incorporated into the grid operations are current global challenges. Hence, buildings must be able to adjust electricity consumption in response to realistic market signals (Farrokhifar et al. 2021). Moreover, it is possible to achieve a more connected and efficient grid operation with the current advancement in smart homes and grid technology in buildings, which account for the larger portion of electricity consumption. The foregoing is a crucial step towards a smart grid implementation (Babar et al. 2020). Integration of RE technologies and energy storage systems (ESS) at load centres is one of the most important features of the smart grid. Thus, having RE system scheduling (power dispatch) and smart control of home energy consumption systems (DSM) based on peak and off-peak periods is suggested (Ma and Li 2020).

### Literature review

The concepts of DSM are focussed on achieving a utility system’s balanced operations (Atia and Yamada 2016). The DSM may be viewed as the implementation of load management at the customer side of the DG utilities according to real-time supply availability. Objectives of DSM implementations in DGs include energy cost reductions resulting from increasing demands and prevention of early failures due to overstretching demands. Such techno-economic goals involve concerns for state policies, regulatory agencies, system operators, utilities and customers. DSM, in a broader concept, consists of demand response (DR) programmes and energy efficiency (EE) (Masters 2004). The DR is a utility-based designed programme for the short-term management of customer demands. The DR programmes provide opportunities for customers to participate in electric grid operations through shifting or reduction of electricity usage for time-based energy rates or financial incentives. Customers are attracted to respond to DR programmes through offerings such as time-of-use (TOU) pricing, critical-peak pricing (CPP), real-time pricing (RTP) and critical-peak rebates (CPR) (Masters 2004). This is essential when considering the BEMS and its strategies for RE efficiency towards real estate development and management, which include residential, commercial, agricultural, industrial and institutional buildings. The importance of emphasising energy management and efficiencies in real estate construction, particularly in building systems, is made by Calvillo et al. (2016).

**Fig. 3:**
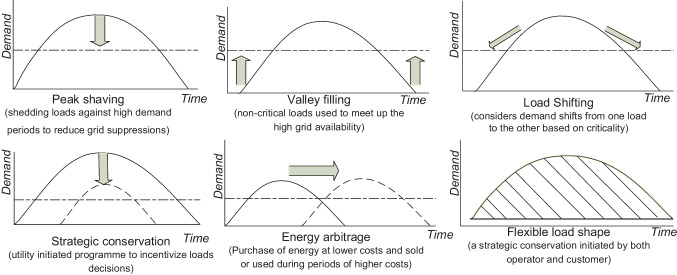
Graphical illustration of demand side management strategies for optimal load scheduling

It is indicated by Debnath et al. (2017), Eze et al. (2016) and Ioakimidis et al. (2018) that DSM is implemented based on clearly defined strategies sketched in Fig. 3. The traditionally used DSM strategies in the literature concerning the applications in RE-based DG systems for load management include peak shaving, valley filling, load levelling and load shifting. *Peak shaving* considers customers’ load shedding as initiated by utility-based DR programmes to release stress on supplies. On the contrary, *valley filling*, as initiated by utility-based DR, tends to raise demands against excess generation to reduce rates of energy curtailments. *Load levelling* is a DSM strategy needed where large fluctuations occur on PDN. Whereas *load shifting* considers the criticality of demand and supply availability to transfer demands among customers or appliances. Uddin et al. (2018) extensively review peak shaving as a DSM strategy concerning the integration of energy storage systems (ESS) and electric vehicles (EV) with the main grid. The traditional DSM strategies, the peak shaving and valley filling are achieved by Ioakimidis et al. (2018) to optimise power consumption profiles in a university building by scheduling the charging/discharging process of an EV parking lot using real-world data of power consumption and parking lot occupancy. Agamah and Ekonomou (2017a, b) developed an algorithm that uses demand profile information and a minimal set of ESS parameters in obtaining an ESS-based operations schedule for demand peak shaving and load levelling. A peak shaving strategy is achieved by Buja et al. (2017) to investigate the capabilities of vehicle-to-grid (V2G)-enabled EVs in executing reactive power compensations through either of the two topologies of a bidirectional battery charger. A valley filling strategy is implemented using rule-based energy management for electric charging from a photovoltaic-to-grid (PV2G) system (Bhatti and Salam 2018). Potentials of load shifting were forecasted to have reduced German-Austrian nationwide electricity expenses by 6% when a 25% DR adoption rate was achieved (Märkle-Huß et al. 2018). Other strategies in DSM include energy arbitrage, strategic conservation, strategic load growth and flexible load scheduling. *Energy arbitrage* is a DSM strategy achieved by energy savings during periods of lower energy costs against periods of higher energy costs. Energy arbitrage is mainly achieved using energy storage devices such as batteries, supercapacitors and EVs. A study by Metz and Tomé (2018) used mixed integer problems to optimise storage dispatch for energy price arbitrage considering time-based differential energy auctions. Likewise, Salles et al. (2016) took advantage of electricity price volatility in 7395 different electricity market locations to improve revenue using energy arbitrage of a generic ESS. Flexible load scheduling implemented using improved TOU pricing methodologies in nanogrid operations achieved a range of 1.72–53.09% consumption cost reduction under binary battery operations (Tijjani et al. 2021).

Strategic load growth, strategic conservation and flexible load scheduling are mentioned to be part of DSM strategies discussed in existing literature such as Jabir et al. (2018), Gaur et al. (2017), Lokeshgupta and Sivasubramani (2018), and Jacob et al. (2018). Like the traditional DSM strategies, *strategic conservation* is a utility-initiated programme that focuses mainly on customer interests in accepting incentives for reduced energy use. *Strategic load growth* is a planned increase in energy sales to improve customer productivity while increasing utilities per kWh energy sales. Whereas *flexible load scheduling* is a programme where customers receive incentives for load building and curtailments. In flexible load scheduling, incentives for load growth and decay are applied to customers interchangeably.

### Study contribution and paper organisation

**Fig. 4:**
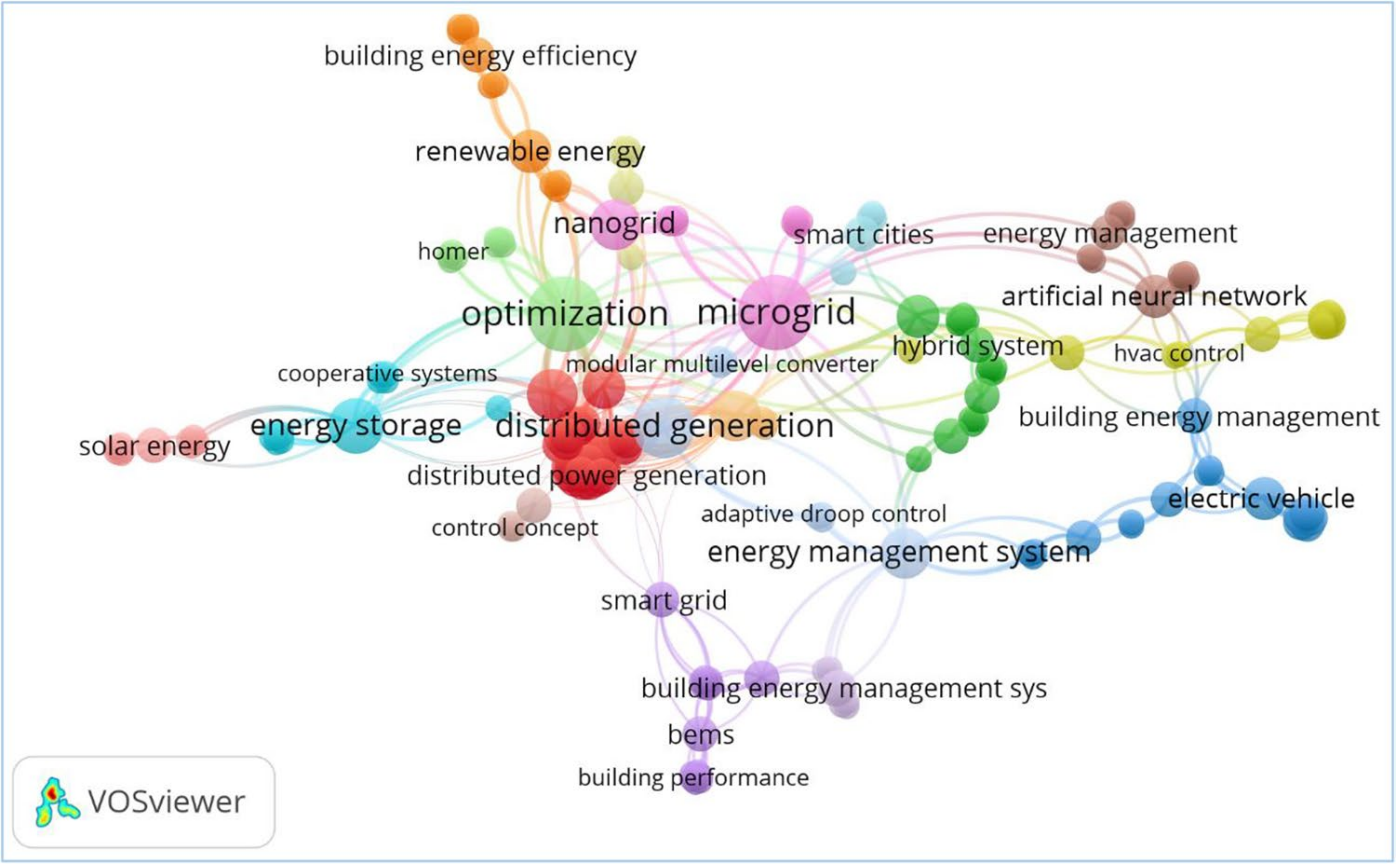
The review’s related areas discussed in the existing literature (VOSviewer)

The foregoing DSM strategies are not prominently used in literature for applications despite their flexibilities and suitability to RE-based DG system problems. This paper analyses literature implementations of the DSM strategies as it applies to emerging DG frameworks and building energy management structures (BEMS). It is indicated in Fig. 4 that the reviewed literature in the areas of DSM and its relationships to BEMS have other major issues of discussion in the paper such as distributed generation/grids (DGs). Classifications of the DGs such as the traditional CGs (in macrogrid and minigrid architecture), the emerging microgrids and nanogrids are extensively discussed. Other related literature consulted and discussed in the paper include optimization techniques, renewable energy (RE) and the impending issues of energy cost. Hence, the paper contributes to the identification of the following points concerning existing literature applications of DSM strategies in DG system developments and building energy management, as highlighted in Fig. 4.

- The DSM strategies implemented based on utility-initiated demand response (DR) programmes are real estate oriented, which covers residential, commercial, institutional and industrial buildings.
- Dump energies due to power generation curtailments are not as economical as it appears. The process may incur additional costs and power losses.
- The real estate’s contribution to energy conservation and emission control could be targeted through the development of nearly zero energy buildings (NZEB).

The rest of the paper is arranged by providing an overview of emerging DG systems based on the perspectives of real estate in the “Distributed grid technologies” section. System planning and operations as the main aspects of the development of DGs are discussed in the “Classifications in distributed grids” section. A detailed review and overview of DSM strategies discussed in the literature is presented in the “Distributed grid planning and operations” section. State-of-the-art applications of DSM strategies used in literature for the development of modern and smart DG systems are discussed in the “Demand side management and strategies” section. An overview of literature perspectives concerning energy management in buildings is discussed in the “Other concepts related to demand side management” section. Implications of climate change on the performance of RE resources and technologies, on the one hand, and seasonal change in customer demands, on the other hand, are discussed in the “Implications of climate change to renewables and energy use in buildings” section. A critical analysis of existing literature regarding research findings with recommendation for further work is provided in the “Limitations and future considerations” section. The paper is concluded in the “Conclusions” section.

## Distributed grid technologies

The need for a study to focus on the development of power grids against the challenges of the twenty-first century is highlighted for the benefits of reliability, efficiency, cost-effectiveness and environmental considerations (Baek et al. 2017). The conventional grid (CG) is a vertically structured centralised system that comprises major units which include generation, transmission, distribution and retail (Ma et al. 2017). Challenges affecting the performance of CGs include the high costs of fuels and the effects of volatile oil markets, transmission losses, carbon emissions and high acquisition costs (Zenginis et al. 2017). Maintaining such systems could be costly in terms of economy and environment (Deckmyn et al. 2017). Other challenges could be reliability due to human errors, natural disasters and transmission losses (Islam et al. 2017; Burmester et al. 2017). Part of the solution to the foregoing problems is to improve supply reliability through increased generation. It is viewed that the power grid’s transmission losses can significantly be reduced by the adoption of modern DG structures, and the need for expansion of the existing structure can be eliminated (Debnath et al. 2017). The DGs are as well good for the support of RE integration to reduce carbon emissions. The DGs also ensure minimisation in the rate of maintenance in addition to fuel and energy consumption cost reduction (Ganesan et al. 2017).

**Fig. 5:**
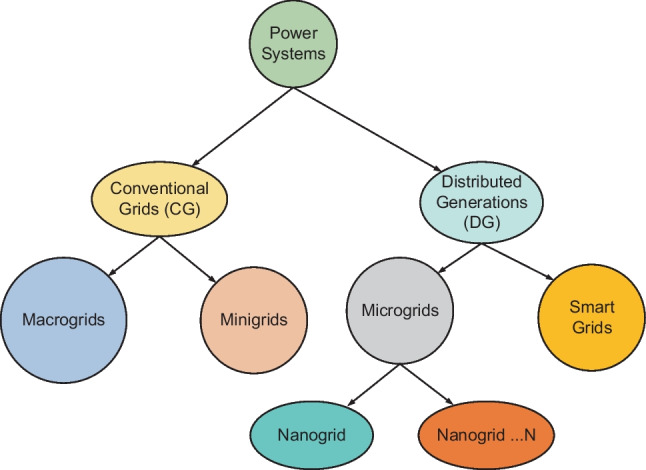
Hierarchies in modern electrical power grids

Apart from the significant impacts of DGs in power systems’ support for RE generation and integrations discussed by Tudu et al. (2019) and Kuang et al. (2016), the REs are good in the harvest of free, abundant and lifetime resources. REs also enable low-capacity generations (microgeneration) for customers to optionally and simultaneously consume and produce electricity in an energy trade-off framework termed “*prosumption*” (Genikomsakis et al. 2017). PV cells, wind turbines (WT) and fuel cells (FC) are a few examples of RE components used as generators in emerging DG systems. The distinctive features of CGs as compared to DGs are represented in the hierarchical diagram of Fig. 5. In the hierarchy, DGs are shown to comprise microgrids and nanogrids as scale-down systems, usually designed for convenience, costs and logistics. Smart grids shown in the diagram indicate structures of DGs as units or subunits equipped with communication facilities. Such systems feature an example of preferred characteristics of a modern power grid highlighted in Table 1.

**Table 1:** Characteristics of CGs, DGs and preferred modern power grids (Masters 2004)

Preferred characteristics of modern power grids	Characteristics of conventional grids	Characteristics of distributed grids
Active customer participation	Customers are uninformed and do not participate	Customers are informed and participate in power generation and energy management
Support for all generation and storage options	Characterised by a centralised generation with no existing storage options	All forms of centralised and distributed generation of different capacities are integrated with plug-and-play facilities
New products, services, and markets	Limited and poorly integrated wholesale markets. Limited opportunities for customers	Well-integrated wholesale markets with openings for emerging electricity markets for customers
Provisions of power quality for the digital economy	Slow response to power quality. Limited power pricing options	Rapid response to power quality and pricing issues
Optimization of assets and efficient operations	Little integration of operational data with assets management	Emphasis on expanded data acquisition of grid parameters and focus on impacts to customers
Anticipation of responses to system disturbances (self-healing)	Respond to protect assets against damages resulting from system faults	Emphasises fault detection, prevention and impacts minimisation on customers
Resiliency against cyber attacks and natural disasters	Slow response and vulnerability to cyber and physical attacks due to human and natural factors	Resilient to cyber and physical attacks and rapid restoration capabilities

## Classifications in distributed grids

Technically speaking, DGs are described as an assembly of different types and sizes of RE technologies such as electrical energy sources, energy storage and consumer appliances linked through power cables, power converters and power control devices as interfaces. There may be no definite topologies in DG architecture, as the composition of components in the system differs depending on planning and operational objectives. However, system capacity and energy demand requirements could be a basis for the classification of DGs concerning components and system sizes. Classifications in DG structures and topologies are usually determined by factors such as geographical location, number and size of buildings, critical demands and bus potentials in the PDNs. Hence, in DG systems capacity-based topologies, the classifications may consist of the macrogrids, the minigrids, the microgrids and the nanogrids.

### The macrogrid

**Fig. 6:**
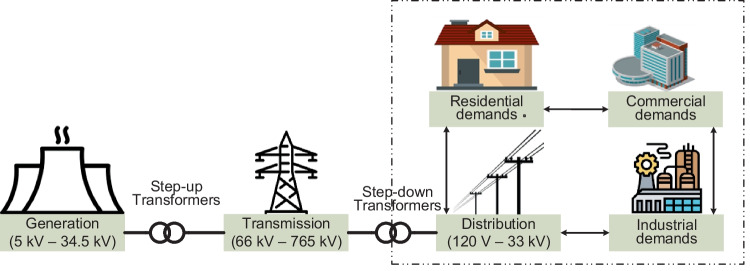
The basic structure of a macrogrid system

Macrogrid topologies are usually centralised systems designed in either ring or radial systems to serve customers within an extensively large area and large population. Generation in this topology is mainly kept at long distances away from load centres. Hence, power transfers from generation to customers are supported through transmission networks, as illustrated in Fig. 6. The architecture in macrogrid topology may be considered as the typical traditional CGs. Voltages handled in this topology are the traditional generation voltages (5 to 34.5 kV), transmission voltages (66 to 765 kV) and distribution voltages (120 to 240 V single-phase and 220/420 V to 33 kV three-phase). The topology is also referred to as the main grid, utility grid or legacy grid. This is the topology dominating larger global economies with heavy residential, commercial and industrial demands.

### The minigrid

**Fig. 7:**
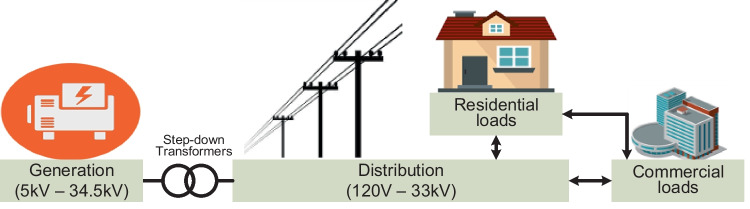
The basic structure of a minigrid system

Factors considered in the design and implementation of minigrid topologies include distance or isolation from macrogrid access, such that service extensions are largely affected by implications of the cost involved. The topology is smaller than the macrogrid in terms of generation capacities and servicing consumer demands. Transmission networks may not be part of this topology as the distance between generation and load centres are significantly close, such that local PDNs are adequate for power transfers. Figure 7 indicates that customers in this topology are mainly residential and commercial. However, this topology is prone to uneconomical and environment-unfriendly generating systems such as multiple diesel plants, biomass or small hydro facilities (Javaid et al. 2018). Generation capacity usually ranges between 1 kVA and 10 MVA in minigrid topologies (IRENA 2018), while it supports low voltages (120 to 220 V) mainly for distribution purposes (Program and ESMAP 2000). Minigrid topologies are mainly designed for developing economies and developed countries with remote locations such as islands. The topologies are known to have high operating inefficiencies and protection issues. Hence, the system is not usually suitable for urban deployments.

### The microgrid

**Fig. 8:**
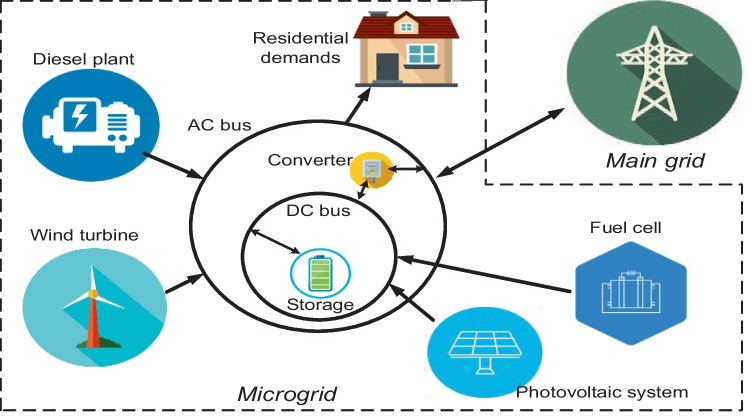
Multi-energy structure of renewable energy-based grid interactive microgrid

Based on the United States (US) Department of Energy (DOE) definitions, the microgrid topology is an electrical entity of generators and loads operating either in isolated mode, in connection with other grids (macrogrids or minigrids) or a network of other microgrids. The system employs either a combination of fossil fuel and RE-based generators. The microgrid topology may not have a definite size; however, the World Bank describes its operating voltages to be below 11 kV (Javaid et al. 2018). Technologies supported in microgrid topologies are highly diverse based on their types and sizes. A microgrid is a typical example of a DG system network with resources sparsely connected, as illustrated in Fig. 8. Issues affecting this topology are complex structures due to the integration of various energy sources and inadequate standardizations. A relatively small market currently affects microgrid prospects to have industry-wide technology standardizations. It is expected that proper standardizations in microgrid systems are necessary to achieve large-scale price reductions and high degrees of interoperability.

### The nanogrid

**Fig. 9:**
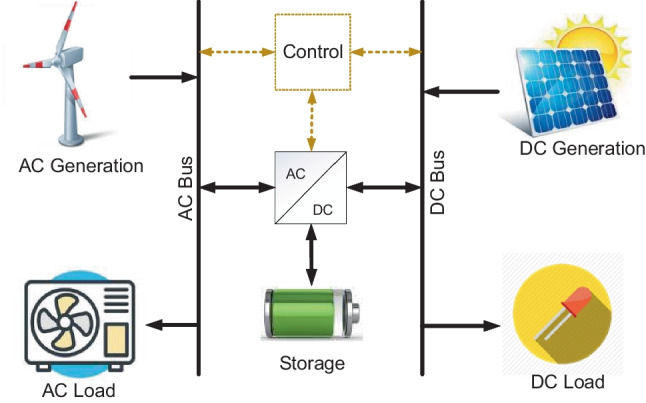
Structures of renewable energy-based AC/DC hybrid nanogrids

Nanogrid is an electrical grid topology that takes a general approach and design principles of microgrids but with much smaller capacity, lower voltage level and lower needs for optimization complexities. A key feature of nanogrid is their ability to interconnect with macrogrids, microgrids and adjacent nanogrids. In similarities with the microgrids, the underlying philosophies about nanogrid concepts are economy (reduction in energy and operational costs, elimination of the cost of macrogrid extension), reliability (increased supply availability), environment (reduced rate of power generation-based emissions) and speed (the reduced time it may take to extend macrogrid services). As obtainable in microgrids, the nanogrid structure illustrated in Fig. 9 operates utilising AC/DC sources and storage. However, it is stated by Nordman (2009) that nanogrids have no concern for a power source. The foregoing view could be considered an outdated concept as any electrical entity without a power source may hardly be autonomous and may be regarded simply as controlled loads within a network of other grid entities. Hence, an entity without a power source may not be qualified for the “grid” affixations. Justifications for the concepts of powered nanogrids are discussed in recent literature where nanogrids are configured to operate in either autonomous or interconnected operations with contributions from nanogrid’s local power sources (Akinyele 2017; Shahidehpour et al. 2017; Moussa et al. 2019; and Cordova-Fajardo and Tututi 2019).

### Smart grids and emerging power networks

The existing electric utility services face challenges such as the need for RE penetration, changes in market dynamics, rapid technological advancements and shifts in customer affordability and tastes while operating the grids. Thus, the foregoing challenges require resilience, accurate forecasting and security against threats both internal and external. The foregoing challenges hence attract unbundling and restructuring in the existing grids and optimising its assets. Comparisons made to distinguish between modern grids and existing grids outline the following aspects needed for the development or transformation of existing grid structures into preferred modern grid architecture referred to as smart grids (Momoh 2012).

- Power system enhancements:expansion and use of REs to offsets the impacts of capacity requirements and carbon emissions.
- Communications and standards: advanced automation and communication systems for the existing power systems are expected to generate a vast amount of operational data to be used in rapid decision-making.
- Computational intelligence: the use of advanced analytical tools for network system power optimizations.
- Environment and economy: customer participation and general enhancement of generation, transmission and distribution networks.

**Fig. 10:**
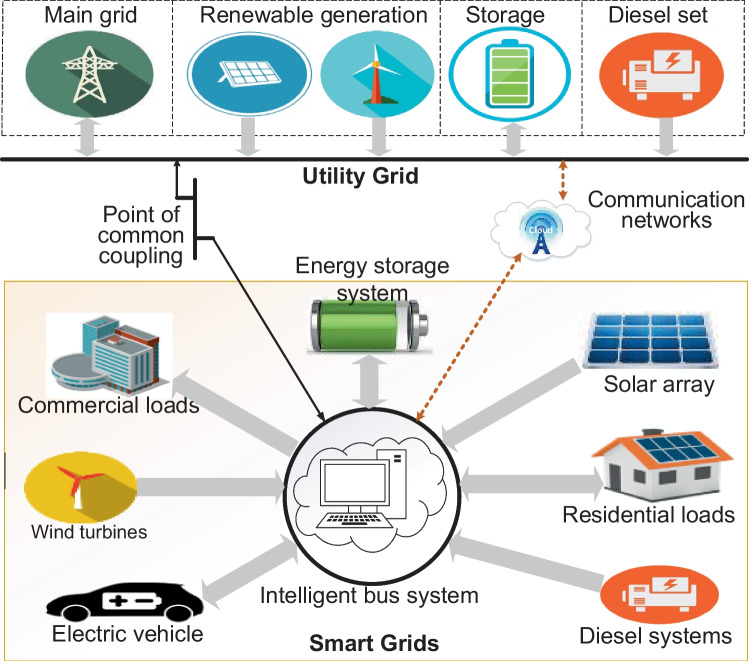
Simplified structure of a smart grid system

Smart grids in Linden et al. (2014) are defined by Kylili and Fokaides (2020) as “an electricity network allowing devices to communicate between suppliers to customers, allowing them to manage demand, protect the distribution network, save energy and reduce cost.” Computational intelligence and communication facilities are the key aspects featured in any smart grid system that are completely alien to legacy networks (traditional grids). This implies that DG topologies such as minigrids, microgrids and nanogrids are classified as smart grids only when the key aspects such as communication facilities and computational intelligence demonstrated in Fig. 10 are included in their design.

Comparative information regarding various types of electricity grid architecture is contained in Table 2. The classifications in the table are made based on the topologies of the grid, the technologies employed, the voltage size handled and the opportunities for deployment. It is worthy of note that the emerging DGs are critical to developments within the real estate sector, as examples are set in the study for nearly zero energy buildings (Martirano et al. 2017).

**Table 2:** Electricity grid features and characteristics

Grid topologies	Supply voltage size	Technologies employed	Deployment	Opportunities/limitations
Macrogrid	66–765 kV	■ Conventional fossil fuel utilisation ■ Energy storage is rarely considered, as demands and supply are highly predictable	■ Extensively large-scale applications such as national grids for industrial, commercial and residential purposes	■ Predictable operations ■ Generation far from load centres ■ Transmission losses ■ Environmental implications ■ High initial and expansion costs
Minigrid	120 V–33 kV	■ Fossil fuel-based systems with the potential to accommodate renewable energy technologies ■ Storage is needed due to the RE system integrations	■ Manly used in moderate applications for rural and urban demands ■ Sometimes used in the electrification of major industries such as manufacturing and mining	■ Predictable performance ■ Close to load centres hence requires no transmission infrastructure ■ Rely mainly on fossil fuels, hence the emission implications
Microgrid	12 V (DC)–220 V (AC)	■ RE-based emerging technologies ■ Microgrids accommodate fossil fuel-based plants such as diesel plants against the intermittence of RE resources ■ Storage is also an important part of the architecture for reliability enhancement ■ Main grid interaction is also supported	■ Community electrification system for residential and commercial applications ■ Suitable for isolated communities in deserts, riverine and archipelagos ■ Special purpose demands such as military formations	■ Clean energy production ■ Distributed loads and generation; hence, no power transmission is needed ■ Complex structures ■ Supports many forms of generation ■ Hardly predictable performance
Nanogrid	12 V (DC)–220 V (AC)	■ RE-based system very identical to the microgrid ■ Differs from microgrids only in size and deployment	■ Limited demands within a building or part of a building that are critical such as data storage management, security system, military services and healthcare systems	■ Clean energy production ■ Distributed loads and generation within a short distance, hence no power transmission needed ■ Low initial cost ■ Simple structures ■ Supports diversified generation ■ Hardly predictable performance
Smart grid	Unspecified (depends on demands, technologies and sizing specifications)	■ Very much the same as the other topologies. However, intelligent communication facilities are used in the system control of smart grids	■ Can be deployed to various applications within commercial and residential settings	■ High coordinated operation ■ Cost-effective performance ■ Intelligent communication facilities render it complex and expensive

## Distributed grid planning and operations

Techno-economic factors such as system reliability enhancement, emission control, renewable penetration, the life cycle cost, the net present cost and the cost of operation dominate objectives in renewable system design. Thus, achieving the foregoing objectives motivates the implementation of schemes for system planning and operations. To highlight the relevance of feasibility and best performance in the implementation of planning and operation schemes, advantages of optimization algorithms are usually taken. However, there are always complexities when handling schemes that involve uncertainties such as the RE resources. Hence, RE resources are characterised by the intermittent performance of generating components, such as PVs, WTs and storage facilities mentioned in the previous sections. This implies that optimal planning schemes are essential to the system sizing and placement objectives.

**Fig. 11:**
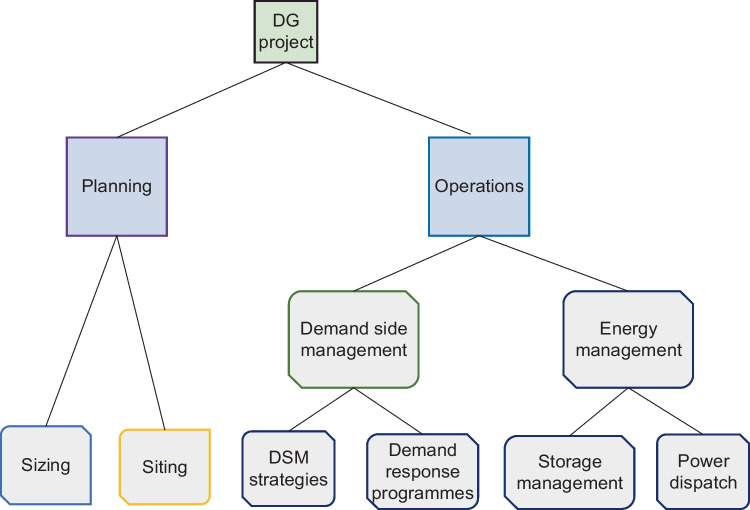
Schemes in DG system’s project planning and operations

The use of RE resources in DG systems considering their resource dispersion, characteristics and intermittence not only affects the systems’ protection and control architecture but also makes it more complex to predict due to size, operational characteristics, cost implications and reliability. Apart from intermittent renewable resources, irregular customer demands contribute to uncertainties in DG performance. Moreover, the intermittent RE resources such as solar insolation and wind speeds affect DG systems’ reliability obliged alternative use of ESS from either a battery as suggested by Strnad and Prenc (2017) and Metz and Tomé (2018), supercapacitor suggested by Zhang et al. (2017) and Sellali et al. (2017), hydrogen storage suggested by Mendes et al. (2016), flywheel suggested by Abazari et al. (2019) and Li et al. (2019) and hydrostatic technologies suggested by Wang et al. (2018a, b). A combination of any two or more of the foregoing energy storage technologies in hybrid form also proved to be effective. Thus, planning and operational coordination in such a multi-source and multi-technology DG system require effective optimization techniques to achieve operational stability and reliability under minimised acquisition and running costs (Li et al. 2017). In Table 3, details of planning and operation schemes are provided to highlight the existing relationships within the hierarchy shown in Fig. 11 about the emerging DGs. The emphases given to the DSM (*front-of-the-metre* activity) strategies and schemes given in Table 3 furthermore clarify the flexibilities of the DSM and its preference for customer support and participation as advantages over the energy management (*behind-the-metre* activity) schemes.

**Table 3:** Highlighting the relationships among the distributed grids’ planning and operational schemes

	Distributed grid life					
Commitment	Initialization		Continuity			
Schemes	Planning (once-off)		Operations (continuous)			
	Sizing	Siting	Energy management(*front-of-the-metre* activity)		Demand side management(*behind-the-metre* activity)	
			Power dispatch	Storage management	Demand side management strategies	Demand response programme
Focus	■ Economic selection of system components for the customer demands ■ Sizing RE-based DG system also considers the location-based RE resources	■ Placement of RE components to optimally harness the project location’s RE resources	■ Optimal scheduling of energy transfers among the trio of power generation, energy storage and customer demands ■ Handling the imminent cases of dump energies ■ Demands can be residential/commercial ■ Demands can be critical or flexible	■ Duplex forms of energy transfer between energy sources and demands ■ DSM strategies such as energy arbitrage utilise energy storage management schemes	■ Optimal load scheduling based on the unit commitments (power dispatch schemes) and customer demands ■ Utilises storage management schemes and demand response programmes ■ Incentives are usually attached to achieve an underlying strategy ■ Dump load management schemes	■ A utility-initiated scheme to optimise demand scheduling ■ Usually design incentives for the DSM strategies ■ An interface programme between power dispatch and storage management schemes, and the DSM strategies

It is emphasised by Loucks and van Beek (2017) that for desired values in system designs and operating policy variables to be attained (such as a complex DG system), identification and evaluation of desired goals and objectives are desired. The goals and underlying objectives are hence achieved using optimization and simulation models. The desired values achieved by suitable optimization methods will lead to the highest levels of system performance and eliminate inferior options.

## Demand side management and strategies

The main objective of power dispatch or unit commitment (UC) in a DG is the economic dispatch of generating components based on supply availability and demand response programmes. Whereas DSM’s main objectives include cost-effective load scheduling based on demand response programmes at the customer end to achieve the following (Attia 2010):

- maintaining a load factor as close as possible to 1.0 and
- maintaining a peak within the proper supply/demand margin.

By achieving the foregoing objectives, utilities could get adequate energy from participating generating units, thereby maximising profits and minimising the per kWh cost of energy. To that effect, traditional DSM strategies such as peak shaving, valley filling, load shifting and energy arbitrage were implemented by Debnath et al. (2017) and Augusto et al. (2017). The DSM was used to minimise energy deficits in a small-scale grid interactive DC microgrid for residential, telecommunications and data purposes (Pannala et al. 2017). Other solutions to energy deficits and stability to demand and supply fluctuations in microgrids were obtained by Molderink et al. (2010a, b). A poor response to peak loads was reported to be one of the limitations of rural RE-powered microgrids. Hence, the use of BESS and diesel plants were suggested to be a good option for solving peak demand issues. However, improper implementation of DSM was discovered to be a serious setback (Augusto et al. 2017). Operational balance was achieved by combining two control methods of prediction and scheduling (Hoogsteen et al. 2016). Primary frequency stabilisation is sought through DR programmes that deploy efficient DSM strategies (Azim et al. 2016). Affine arithmetic is a proposed model for load forecast that minimises uncertainties in demands (Avila et al. 2015). A shift from the known centralised control type of DSM was proposed by (Balakrishnan et al. 2017) using an agent-based method for efficiently balancing supply and demand. The DSM is known to be implemented based on suitable strategic schemes. An example of a peak clipping strategy applied in literature is a successful trade-off achieved by Nunna and Doolla (2013). In Amrr et al. (2018), reliability and economy-centred power management and load scheduling were activated using two control schemes of mode selector and source selector. A standalone nanogrid based on PV only was designed for a residential house using a Middle East climate. Load calculations for sizing of batteries and PVs, power consumption, control and monitoring were done by Akmal et al. (2016). Saini (2007) categorises DSM into the following three (3) activities upon which implementation strategies may be derived and discussed.

- Energy demand reduction programmes. This is an activity where demands are reduced through better and more efficient processes such as smart energy buildings or the use of energy-efficient equipment.
- Load management programmes. This is an activity of changing load patterns through demand shifts and demand curtailment during peak periods and peak rates.
- Load growth and conservation programmes. This is an activity for change of load pattern through substitution or deferment of loads.

**Fig. 12:**
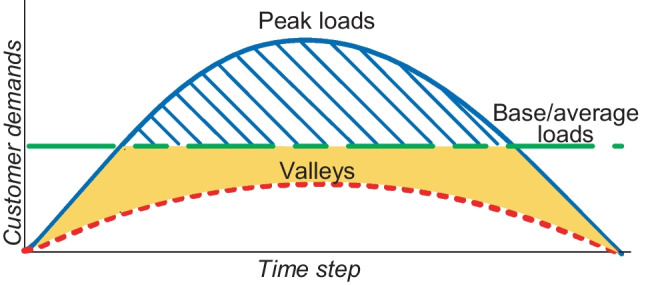
Demand side management strategy curves

In summary, DSM is defined as a programme designed to modify customer use of electricity for energy and cost-related savings. The DSM programmes are usually prepared to control energy consumption on the customer side of the metre (*behind-the-metre*). Many strategies used in DSM applications are designed to reduce dump energies (energy wastages), curb energy consumptions (in shiftable loads) during periods of low electricity supplies, reduce energy costs and minimise the system’s costs of operations. There are many strategies designed or adapted in literature for effective DSM implementations. Examples of such include a study prepared and presented by Debnath et al. (2017) to investigate a wide range of DSM strategies whose demand curves are illustrated in Fig. 12. The investigated strategies were meant to be used in enabling customer participation and management of DGs as one of the preferred characteristics of emerging modern power grids.

### Peak shaving

Peak shaving also known as peak clipping is a traditional DSM strategy that considers cutting off a portion of loads at a time in peak hours of electricity demand without adverse effects on overall demand curves. The benefits of peak shaving are identified by Molderink et al. (2010a, b) as a solution to varying daily electricity needs, clean energy production and additional unpredictable loads such as EVs. There are examples of peak clipping implementation in the literature. Peak clipping is achieved through the modification of a reference load by applying a cap for maximum power that can be delivered on request (Augusto et al. 2017). Simulation results by Martirano et al. (2017) show that a reduction of peak power by 12% and average power of 23% was achieved in a smart microgrid equipped with heating and domestic hot water. A *selective* peak clipping is proposed by Nunna and Doolla (2013) to achieve energy balance in microgrids. Battery power is optimally synthesised over a specified period to achieve peak shaving and reduction in grid energy buffering (Serpi et al. 2017). Prototype control methods were used to validate the simulation results of a microgrid connected to the grid and in island modes for peak demand minimisation and diminishing of stress on a CG network (Pannala et al. 2017). The advantages of peak shaving to power utilities, according to Uddin et al. (2018), include a reduction in per kWh electricity generation cost. Likewise, customer benefits from corresponding per kWh energy cost reduction as utilities normally transfer the economic burden to customers. The major disadvantage of peak shaving is a breach in customer comfort.

### Valley filling

There are periods when low demands are experienced regarding base loads. Higher levels of power generation during such periods may comparatively be at a loss if demands are not raised due to unused (dump) energies. Considering the RE type of power sources in this scenario and the costs of energy involved, generation curtailment may likely not be an optimal decision. The use of storage and flexible (transferable) loads are examples of methods of achieving valley filling. An example of a valley filling strategy was achieved by Augusto et al. (2017) by restricting flexible loads to off-peak demand scenarios. Valley fillings were also realised in a study that proposed charging an EV from a PV/grid system using a rule-based EM system (REMIS) (Bhatti and Salam 2018). To regulate power consumption in a building, vehicle-to-building (V2B) concepts were formulated to achieve peak shaving and valley filling through mathematical modelling in MATLAB (Ioakimidis et al. 2018). Stochastic algorithms with Monte Carlo simulations were used to stabilise the grid through valley filling strategies (Nazarloo et al. 2018). A binary programme was used to achieve desired aggregate load profile through valley filling (Sun et al. 2016). A centralised charging is proposed by Liu et al. (2017) to be applied as a method of flattening the demand profile through valley filling for overnight charging. Advantages of valley filling strategies to utilities include a reduction in dump energies and the burden of generation curtailments are avoided. Customers may as well benefit from valley filling through flexible loads that are normally shifted to periods of lower per kWh energy costs.

### Load levelling

In power distribution networks where large fluctuations of loads are experienced, load levelling is the most appropriate DSM strategy required to be applied. In load levelling strategy, efforts are ensured to reduce differences between the highest and lowest values of demand profiles (Agamah and Ekonomou 2017b). In literature, diverse load levelling applications were implemented as an important EM strategy for the reduction of losses and stability of DGs. Maximum power point tracking (MPPT) controller was used in [47] to improve energy storage and load levelling. In a study presented by Agamah and Ekonomou (2017a), load levelling objectives were combined with objectives of optimal ESS and peak demand schedule. Multiple benefits of energy storage were reviewed by Nikolaidis and Poullikkas (2018), which include load levelling as a DSM strategy. The load levelling was also realised through integrations of EVs into smart grid systems, where frequency regulations and other ancillary services were found to be beneficial to utilities (David and Al-Anbagi 2017). In Buja et al. (2017), reactive power-based load levelling was also implemented. Reducing energy costs and protecting consumer privacy was achieved by Chin et al. (2017). Resources such as ESSs and EVs are mainly used in load levelling implementations, thereby eliminating the need for network expansions.

### Load shifting

In load shifting strategies, load shedding or load building synonymous with peak shaving and valley filling, respectively, at a particular point in time are not considered. Rather, the strategy considers the transfer of loads from one appliance’s demands to another based on the criticality or flexibility of loads and periods of supply availability. Examples of load shifting discussed in the literature include investigations into the potentials of peak shaving, valley filling and load shifting (Zhang et al. 2015). Load shifting strategies were applied in the sizing and selection of ESS for a PV/wind-powered DG distribution system (Sepulveda et al. 2018). The benefits of load shifting were analysed while optimising generator and load sizing schemes in a standalone microgrid (Akram et al. 2018). A day-ahead load shifting is considered while investigating the benefits of DSM to both the utility and customer sides of the DG. There were classifications of PV users based on pre-grids and post-grids terms, where the latter recorded energy savings through load shifting activities and sufficiency attitudes (Wittenberg and Matthies 2018). Load shifting was proven to increase levels of self-reliance in terms of energy consumption for a heat pump coupled with PVs (Romaní et al. 2018). An achievement of up to 6% reduction in nationwide electricity costs was recorded, as claimed by Märkle-Huß et al. (2018). The load shifting strategy was used in the reduction of peak demand and reshaping of a load profile in the proposed multi-objective optimization framework (Bastani et al. 2018). It is worthy of note that load shifting as a strategy is feasible only when load classifications are made based on demand’s criticality and flexibility rankings.

### Energy arbitrage

Energy arbitrage is described as energy vending at the time of higher energy prices after a stored purchase at lower prices. Energy arbitrage may also be considered as efficient storage of energy during excess production for use at the time of low or loss of power supplies. This strategy is mostly suitable to REs based or RE-dominated DG technologies. Energy arbitrage is usually achieved using efficient storage systems that can be BESS, pumped hydro, supercapacitor, compressed air, hydrogen storage and flywheel (Nikolaidis and Poullikkas 2018). Examples of energy arbitrage implementation in the literature include a study presented by Salles et al. (2016) which considers reducing costs and improving the energy efficiency of generic model ESSs. Mathematical modelling is used to determine the feasibility of investing in energy storage of vanadium redox batteries, which recommended optimization to 75% for energy arbitrage to be profitable (Coronel et al. 2018). Energy arbitrage was listed among the numerous benefits of using ESS in electric power grids (Nikolaidis and Poullikkas 2018). A study presented by Cui et al. (2017) considers what was referred to as *an extended-term* energy storage (ES) arbitrage problem through a bi-level ES arbitrage solution. A portfolio theory-based approach was also proposed to achieve optimization of energy storage capacity share in different energy markets. Hence, frequency response and congestion costs were proposed for distributed network operator (DNO) markets (Yan et al. 2018). A sensitivity analysis was carried out to ascertain the price volatility required to generate profit from energy arbitrage operations (Metz and Tomé 2018). A community LV distribution system equipped with ANFIS was used for voltage management, energy arbitrage and peak load reduction, respectively (Wolfs et al. 2018). The increased complexities due to increasing penetration of REs in power systems were reduced by increasing storage requirements at the LV network that calls for stochastic integer linear programming for the solutions (Touretzky and Baldea 2014). Energy arbitrage using ESS may be used as dump loads against implications of excess generations. However, dump energies may persist with ESS fully charged. The cost of managing stored energy in ESS during charge/discharge actions against losses is yet another challenge.

### Strategic conservation

Strategic conservation is a consumer-centred DSM strategy that usually originates from utility-based DR programmes specific to changing power usage patterns. Incentivised sales and usage reduction change load shapes in the programme. It is pointed out by Khan (2019) that strategic conservation is achieved by making efficient use of energy or by reducing the amount of energy service. This clearly defines strategic conservation of energy as an effort to reduce consumption by using fewer energy services. Although Kumar and Harish (2014) insisted that for strategic conservation and other DSM strategy implementations to be successful, a demand forecast that defines how electricity is consumed shall be prepared. It is found in the literature that a control technique is applied by Romaní et al. (2018) to reduce energy import for load shifting and load conservation strategies against peak periods. It is worthy of note that with differing customer tastes and behaviour, demand forecasts tend to be difficult.

### Strategic load growth

This is a planned increase in energy sales ahead of valley filling strategies due to the utilisation of smart power technologies such as EVs, automation and industrial process heating. This strategy aims to increase the market share of loads that sometimes involve the addition of new customers. Strategic load growth programmes are aimed at improving customer productivity while increasing utilities per kWh energy sales. Strategic load growth is similar to valley filling, but the level of sales in strategic load growth is greater. A lot has been mentioned the strategic load growth in literature such as Debnath et al. (2017), Eze et al. (2016), Attia (2010), Kumar and Harish (2014), and Al-enezi (2010), and without any known research implementation undertaking. Advantages of the strategy include minimisation of dump energies and energy cost savings. The method could however be feasible only to systems with efficient dump loads such as energy storage systems and in cooperation with another DSM strategy such as valley filling and load shifting.

### Flexible load shapes

This is a programme where customers receive incentives for load curtailments as a result of deterioration of reliability or quality of service. The DSM programme by the utility deviates from permanently sticking to a specific load shape, such that incentives attached to load growth and decay on the customer sides are applied interchangeably. Like strategic load growth, the flexible load shape is another DSM strategy whose concepts are largely mentioned, but without known research implementations in the literature. The traditional power system structures (CGs) may not find flexible load shape strategy as convenient for applications due to highly predictable power generation patterns that tally with the customer demands most of the time. However, emerging DGs operating under highly intermittent RE generations require smart DSM strategies such as flexible load shapes, such that customer demand curves may be shaped based on supply availability, energy cost and customer demand fitness (energy cost affordability). Hence, the comforts of customers need to be pegged on the fitness functions of every individual customer (Tijjani et al. 2021). The flexible load shape strategy is envisaged to improve the autonomy of a DG system interacting with the main grid. However, the flexible load shapes may not be suitable in standalone systems where unified tariff regimes are applied.

## Other concepts related to demand side management

It is established that the techno-economic objectives in energy systems projects cover both planning and operation schemes. The operation schemes mainly consider EM strategy applications for optimal power dispatch, storage management and the application of DSM strategies. Power dispatch is strategic to energy system operations. However, power dispatch may be more strategic in the operations of an RE-based DG architecture due to the diverse technologies and intermittent resources involved. Hence, consequential reliability challenges are expected in DG operations such that what matters most to the system’s optimal operations are implementations of DSM strategies. The DSM is known to be flexible enough to be made to follow supplies in the event of intermittent power dispatch. In the implementation of power dispatch, storage management and DSM schemes, optimization tools and methods are found to be mostly useful. Optimization as a concept is designed to minimise or maximise the output parameters of a system by optimal selection of input parameters (Insam 2017). Achievements in realising the main techno-economic objectives which include capacity reduction, cost minimisations, profit maximizations, energy consumption reductions, emission mitigations and efficiency enhancements emphasise the importance of optimization methods to the initialization and management of energy system projects.

### Optimization concepts and algorithms

Optimization is a household name in almost all fields of human endeavour, engineering, business development, industrial activities, internet routing and holiday planning. The main focus of optimizations in energy systems covers minimisation of costs, reduction in energy consumptions, time resource management, maximisation of profits, increased outputs, improved performance and better efficiencies (Yang and He 2016). Energy system applications are highly analytical that demand mathematical programming in finding solutions to real-world problems. In energy system optimizations, computer simulation tools are used in either *user-designed software* or commercially available off-the-shelf *application software*. Whatever software is considered for implementations in energy system optimizations, an algorithm or step-wise guidelines define the method upon which problem solutions are achieved.

1 $$\mathrm{Minimise}\; {f}_{i}\left(x\right), \;\;\;\;\;(i=\mathrm{1,2},3\dots \dots \dots \dots \dots \dots M)$$

2 $$\begin{array}{cc}\mathrm{Subject\; to}\; {h}_{j}\left(x\right),\;\;\; (j=1, 2, 3\dots \dots \dots \dots \dots \dots N)\end{array}$$

3 $${g}_{k}\left(x\right), \;\;(k=1, 2, 3\dots \dots \dots \dots \dots \dots P)$$

4 $$\mathrm{where}\; x=({x}_{1}, {x}_{2}, {x}_{3}\dots \dots \dots \dots \dots {x}_{d})$$

Optimization is defined by Insam (2017) as a method of economic selection of inputs for a system or process to achieve the best and yet feasible outputs based on underlying techno-economic constraints. Optimization must have a carefully defined algorithm for its implementation. In mathematical terms, most optimization problems are written in generic forms given by Eqs. (1)–(4), where ${f}_{i}\left(x\right)$, ${h}_{j}(x)$ and ${g}_{k}(x)$ are functions of the design vector and ${x}_{i}$ of $x$ are design variables that can be real continuous, discrete or a mixture of the two. The choice of optimization algorithms depends on what problem is on ground to solve. Hence, Yang and He (2016) stresses that the following options and relevant questions need to be understood for a proper choice of optimization algorithms to be meaningful.

- For a given type of problem, what is the best algorithm to use?
- For a given algorithm, what kind of problem can be solved?

**Fig. 13:**
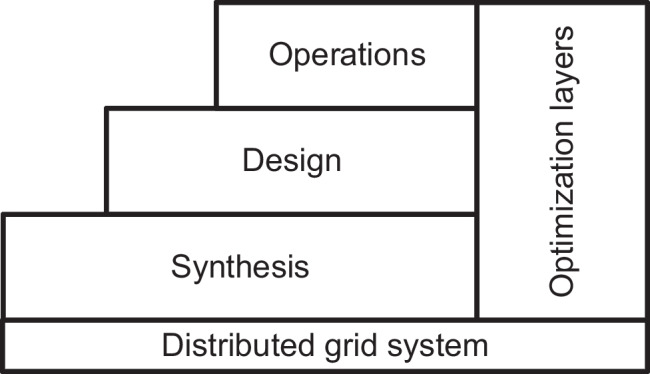
Optimization layers considered in the DG project

The two statements and corresponding questions may not be easy to comprehend as they sound. However, it is important to understand that many problems can be solved using a particular algorithm, as there are many efficient algorithms to use in solving particular problems. It is worthy of note that the choice of algorithm largely depends on the expertise of a designer (decision maker) based on the types of the problem on the ground and available resources. Other factors that may be considered in the choice of optimization algorithm are computational costs, software availability and time constraints for a solution to be achieved. In Urbanucci (2018), optimization concerning technology-based applications is implemented in a three-layered hierarchy. The synthesis, design and operations are shown in Fig. 13.

### Demand response programmes

The main objective of DSM in RE-based DGs is to, as much as possible, maintain a real-time balance between energy production and customer demands. Thus, DSM implementation aims to reduce energy costs resulting from increasing demands that also overstretch existing utilities. Such energy cost reduction goals include factors that involve concern for governments, system operators, utilities and customers. Other factors include environmental objectives such as emission reduction. The DSM, in a broader concept, consists of demand response (DR) programmes and energy efficiency (EE). DR is described as a designed programme for the short-term management of energy demands on the customer side of the electricity network. The DR provides an opportunity for customers to participate in electric grid operations through shifting or reduction of electricity usage for time-based rates or financial incentives. The DR programmes are designed to lower retail rates. Customers are attracted to respond to DR programmes through offerings such as time-of-use (TOU) pricing, critical-peak pricing (CPP), real-time pricing (RTP) and critical-peak rebates (CPR). The DR programmes also provide the opportunity for direct load control of heavy appliances such as air conditioners and water heaters. In preferred modern power grids, automatic switching is employed in diverting or reducing power in strategic places to avoid overloads and power failure. Advanced metering infrastructure is used to expand the range of time-based rate programmes. These programmes also have the potential of reducing peak demands that save utilities from power production and management costs, where expansion and extension of existing infrastructure are deferred.

### Standard residential tariff system

In standard residential tariffs, billings are prepared based on monthly kWh consumed in addition to metre and equipment charges. The kWh electricity is the actual energy consumed by customers, whereas metre charges cover expenses incurred while installing and maintaining electricity metres. There are two structures upon which standard residential tariffs are formed. The *inverted block rate structure* refers to billings based on tier blocks. The billing system considers the initial tier with lower rates for kWh consumed initially within a certain tier block. The subsequent tier blocks are charged with rates higher than the preceding tier blocks. This structure of standard residential tariff is designed to discourage excessive consumption for energy conservation purposes. The other tariff structure is based on *declining block rates*. The structure is opposed to energy conservation and makes electricity cheaper as customer demand increases. The two structures are usually applied interchangeably to manage the usage of electricity during the summer and winter seasons.

### Residential time-of-use tariffs

Residential time-of-use (TOU) tariff structures are designed to persuade customers to shift loads away from peak demand periods. Demands for electricity usually increase based on seasonal factors. During summer seasons, demands are raised in the afternoon for loads such as highly rated air conditioners for space comforts. Peak demand periods during winter are experienced for space heating. Hence, such periods of higher energy demands are managed by the application of TOU rates such that more charges for electricity bills are recorded. In other words, TOU rates/charges are much higher for electricity usage during *on-peak* periods. An example set by Masters (2004) indicates that TOU rates are more costly than the standard rates. Based on the foregoing examples, conventional TOU resembles standard tariff’s *inverted block rate* and *declining block rate* structures, with a more adverse effect on customer interests. In other words, conventional TOU is more economically favourable to the utilities. However, TOU provides opportunities for customers to generate, use and export electricity to utility systems through *net metering*. Customers with roof-top PVs have opportunities to escape the need for expensive utility-supplied *on-peak* electricity. In this case, monthly net electricity consumed or generated is billed or credited to the customer at applicable TOU rates.

### Real-time electricity pricing

Real-time pricing (RTP) is described as the ideal structure for electricity pricing where the true cost of electricity is reflected in rates that constantly change throughout the day on a daily basis. The RTP structure of electricity pricing is offered by utilities based on day-ahead, hour-by-hour real-time pricing. This proposed tariff structure is viewed as an improvement over the TOU structure that attempts to capture the true cost of utility service. The TOU is viewed to be relatively crude since they only differentiate between a structure of a large block of periods such as on-peaks, off-peaks and partial-peaks. The RTP may require advanced smart metering equipped with effective communication facilities. RTP may also swing larger customers’ use of electricity periodically, a case that may lead to highly overstretching demands at one time and high dump energies at the other.

## Implications of climate change to renewables and energy use in buildings

The RE resources are certainly dependent on climates, which vary significantly with time and region. The climates are known to be susceptible to changes, where methodologies in existing literature were used to assess or estimate the implications on RE performance and customer demands such as the smart buildings. This motivated a review to assess projects’ quantitative estimates of climate change that affect RE technologies such as solar, wind, hydro, biomass and fuel cell. The effort was to address the economic estimates and value chain based on existing gaps within the RE technologies and certain geographical regions (Solaun and Cerdá 2019). Errors were observed in RE capacity (planning) and production (operations) among the United States and European countries, where attractive policies and further research were suggested to achieve reliable RE technology and accurate weather predictions (Al Irsyad et al. 2019). Gernaat et al. (2021) considered an integrated assessment model to estimate the effects of climate change on eight renewable technologies across warming scenarios.

In addition to the implications of climate change on the planning and operations of the RE supply system, the demand side of the utility is also affected. Buildings, as examples of the demand side entities, require designs for efficient energy utilisation. Hence, Cabeza and Chàfer (2020) systematically explored technological options and strategies towards zero energy buildings. Zhai and Helman (2019) objected to the existence of several models for the assessment and mitigation strategies on climate change without a narrow prediction of its influence and identified four models accurate enough to predict the potential energy implications of climate change in a campus building stock. A statistical and dynamical down scaling method for the investigation of the energy demands for space comfort in buildings was utilised (Berardi and Jafarpur 2020). Flores-Larsen et al. (2019) evaluated the impacts of climate change on the energy performance in residential buildings by simulating a compact mid-income house using EnergyPlus. The software was used to analyse whether bioclimatic strategies were appropriate for the design of future buildings.

Building energy management systems (BEMS) are currently utilised to manage electricity utilisation in buildings. BEMS is defined as a set of tactics and procedures used to increase the performance, efficiency and energy utilisation in a system (Bonilla et al. 2018). BEMS approaches are divided into two categories, the active and the passive. The passive approach is based on offering future strategies and increase in the user’s energy awareness to indirectly influence and minimise the consumption of energy in buildings. The passive approaches are sometimes achieved using incentives. Active techniques are based on a combination of the actuators and sensors infrastructure that exists in a structure. Using smart building actuators and devices to regulate energy waste scenarios, they rely on lowering energy waste levels in their environments (Degha et al. 2019). It is worthy of note that communication facilities are always essential in the implementation of active approaches.

### Limitations and future considerations

Reliability, stability and economy are the broad objectives usually attained in optimal DG system planning and operational design goals, as summarised in Tables 4, 5, and 6. The major classifications in DG operations were the power dispatch and the DSM. While appreciating the advantages of the DSM over power dispatch in RE-based systems, Kylili and Fokaides (2020) emphasise better DSM implementations through flexible load management. In recent studies, traditional strategies such as peak shaving, valley filling, load shifting and energy arbitrage were implemented, as exemplified by Augusto et al. (2017), Hossain et al. (2018), Martirano et al. (2017), Yaghmaee et al. (2017) and Fernandez et al. (2018). However, implementation of the foregoing strategies affects customer comfort and may not be consistent with the stochastic RE generation and customer demand patterns at all times. Hence, the case may lead to a high rate of dump energies and unmet demands. Moreover, energy curtailments may not be an optimal option as the process incurs additional losses and increased operational cost implications. Hence, optimal load management is appropriate for coordinating customer demands based on real-time supply availability. In terms of emission control, there may also be the need for intensive research in real estate to focus on the development of NZEBs, as demonstrated by Martirano et al. (2017) and Galisai et al. (2019).

**Table 4:** Critical analysis of DSM implementation methods in the literature

Ref	Year	Components	Problems/study objectives	Algorithms	Achievements	Shortcomings/further studies
Martirano et al. (2017)	2017	• PV • CHP • Thermal storage	To develop “a virtuous and flexible load profile” for nearly zero energy building (NZEB)	Building energy management system (BEMS)	12% peak load was reduced without the full cooperation of the residents 23% of average power consumption was reduced without the full cooperation of the residents	Only self-consumption is supported. No plan for grid exports
Yaghmaee et al. (2017)	2017	• PV-based multiple DGs	Optimizations for both customer and utility costs	Two-tier cloud-based DSM	Customer consumption costs were reduced The peak load and PAR of the power grid were improved	The proposed systems need high computation and large storage for customers’ data
Yang et al. (2018)	2018	• WT • PV • BESS • PEV	Optimal online demand response programme for residential microgrids	Distributed online algorithm	There were successful optimizations for costs and power balance using a price-based incentives approach The proposed method achieves system management coordination with uncertainties	Complex computations are needed to analyse the system performance feasibilities The authors suggested the use of sliding mode control and fuzzy techniques to improve the handling of uncertainties for both RE generators and load conditions
Fernandez et al. (2018)	2018	• RE powered DERs	Cost savings for the consumer and peak average ratio (PAR)	Game-theoretic DSM	Cost and reductions were realised (1.76 and 1.81 for summer and winter, respectively) Peak-average-ratio (PAR) reductions were realised (9.17% and 9.68% for summer and winter, respectively) This development saved customers from the installation of ESS	Behavioural patterns of consumers were not taken into account in the proposed algorithm; hence, there is a risk of compromising real-life scenarios, which may have to do with comforts, etc The work falls short of operational scenarios; hence, real-world solutions may not be realised The work might need to consider residential and industrial systems separately for better results The algorithm encounters communication failures
Javaid et al. (2018)	2018	• PV • Wind • ESS	Efficient scheduling of loads and integration of resources	Home energy management control system using binary PSO	In the proposed scheme, (i) voltage rise due to high RE integration is avoided; (ii) COE is reduced; and (iii) PAR is also reduced for the aggregated load	Results indicate that energy trading consumers must be large for the storage capacity to be reduced. In other words, large integration of RE must either be solved by exports or large storage capacities or both
Kou et al. (2018)	2018	• Wind • ESS	Energy schedule to contain the effects of uncertainties of RE generation and loads	Convex quadratic programming and machine learning and MPC	Gaussian and non-Gaussian uncertainties of the RE resources and loads were efficiently handled	There was no consideration for controllable loads. Hence, the proposed work did not consider DSM, which is key to achieving the scheduling objectives of the present work
SoltaniNejad Farsangi et al. (2018)	2018	• PV • WT • CHP • FC • PEV/TESS	Operational cost reduction for a price-based and incentive-based DSM	Two-stage MILP optimization	Load shifting of 10% and 20% show the operational cost of case 2 over case 1 of $123.2819 and $246.4966, respectively	There was an increase in operational costs of $8267.8394 in the islanded mode of microgrid operation It is suggested for future work; (i) an improved method to achieve multi-objective economic, emission and planning for the system energy management (ii) methods of reducing transmission losses in the PDN
Thomsen (2018)	2018	• PV • CHP • BESS	Operation planning to achieve a minimal cost of operation	Mixed integer problem in GAMS	Storage availability is prosperous, where reserve markets are considered Significant revenue is realised even with limited generation capacities DSM implementation ensures the benefits of price spread at the spot market	The work lacks an integrated planning model, which is meaningful to the system’s operational characteristics There were many operational cost variations among operational scenarios
Oh (2018)	2018	• PDNs	Cost minimisation and social welfare maximisation	Willingness-to-pay WTP-DSM mechanism	DSM implementation decreases demand that minimises the cost of generation	The demand decrease is limited to 10%. This is not the optimal limit. However, the search for an optimal solution must consider the relative effects on social welfare
Majidi and Zare (2018)	2018	• PV • Wind	Optimal economic operation of smart energy hubs (S.E. hubs)	Scenario-based information gap decision theory (IGDT)	Under technical constraints, SE hubs were integrated into PDNs Modelling of uncertainty-based load, price and RE units Economic operation and uncertainty-based performance of S.E. Hubs are strengthened under DSM programmes	The grid purchases in all cases of optimization are high. This can be the most viable way of utilising the RE resources
Monyei and Adewumi (n.d.)	2018	• Thermal • Power • plant	To harmonise demand and supply constraints through (i) reduction of electricity costs, (ii) reduced operational costs and (iii) reduced emissions	Standard deviation biassed genetic algorithm (SDBGA)	The customer-initiated dispatch achieves a lower cost of energy than the utility dispatch initiatives The utility-initiated dispatch achieves a better minimised DSM window, lower operating costs, higher plant capacity utilisation and a more evenly distributed profile	Most of the achievements are gained for utility-initiated dispatch. How then can a customer-initiated DSM strategy maintain the COE achievement and further achieve minimisation of DSM windows The author proposed a multi-window within a dynamic pricing scheme
Lokeshgupta and Sivasubramani (2018)	2018	• Thermal • Generating plants	Investigates the benefits of DSM to the generation side	Combined multi-objective economy and emission dispatch (MODEED) with DSM using MOPSO	The results obtained show that benefits were realised to both utility and generation, mostly at 20% participation	The study proposes extending optimization models to the microgrid environment
Bastani et al. (2018)	2018	• Smart grids	Reduction of peak load demand and reshaping of the load profile for SGs to achieve the following: (i) cost minimisation, (ii) GHG emissions and (iii) customer satisfaction	$$\delta$$-constraints multi-objective optimization	The proposed method succeeded in meeting the desired load curves while obtaining a significantly larger pareto frontier solution set in less computational time	Risk assessment should be applied to evaluate the efficiency of the generated solutions It is suggested that the operation planning of SGs to consider load shifting and interruptions can improve the quality of the load scheduling
Craparo and Sprague (2019)	2019	• PV • Wind • Diesel	Optimal scheduling of generations and loads in military smart microgrids	Rolling horizon optimizations	The achievements involve both supply and demand sides for energy management, unlike reference work Results indicate a significant in fuel savings without affecting the system performance	Results obtained indicate insensitiveness to storage capacity, storage efficiency, generator run and rest times Time-shiftable loads were suggested to improve optimization potentials
Arasteh and Riahy (2019)	2019	• Wind • ESS	Optimal operation of the market-based wind system	MPC	There were cost savings of 12.18% and 6.3% against 1st and 2nd control approaches There was up to 13.9% and 4.9% daily energy utilisation as well	It is recommended that, for future work, appropriate selection of the prediction and control horizon of the MPC There is a need for data management for a large-scale power system

**Table 5:** Demand side management strategies discussed in the literature

DSM strategies	Reference	Features	Merits	Demerits	Remarks
Peak shaving	Ioakimidis et al. (2018), Uddin et al. (2018), Agamah and Ekonomou (2017a, b), PANNALA et al. (2017), Augusto et al. (2017), Nunna and Doolla (2013), Molderink et al. (2010a, b), Martirano et al. (2017), Serpi et al. (2017), Wang et al. (2018a, b), Mallol-Poyato et al. (2016)	Trimming off a portion of energy consumption in periods of higher demands to avoid overstretching supplies	■ Solutions to varying daily electricity needs ■ Reduction in per kWh energy cost	■ Economic burdens are normally transferred to customers ■ Customer comforts are comforts breached	Mostly suitable to systems with highly predictable operations such as vertically structured conventional grids
Valley filling	Ioakimidis et al. (2018), Bhatti and Salam (2018), Augusto et al. (2017), Saini (2007), Nazarloo et al. (2018), Sun et al. (2016), Liu et al. (2017), Al-enezi (2010), Guelpa et al. (2019), Pan et al. (2019), Mortaz et al. (2019)	Building up demands during periods of high power generation	■ Dump energies are considerably reduced ■ Burdens of energy curtailments are removed ■ Customers often benefit from the low cost of energy	■ Imminent use of storage facilities ■ Load classifications are the order of criticality and flexibility needed	■ With valley filling, energy losses are avoided ■ Customer comfort is jeopardised
Load shifting	Märkle-Huß et al. (2018), Lokeshgupta and Sivasubramani (2018), Augusto et al. (2017), Zhang et al. (2015), Sepulveda et al. (2018), Akram et al. (2018), Wittenberg and Matthies (2018), Romaní et al. (2018), Bastani et al. (2018), Yaghmaee et al. (2017), Kumar et al. (2019), Tu et al. (2019)	Efforts to reduce differences between high- and low-demand profiles	Reduces the need for system upgrades or expansions	Mostly beneficial to utilities	Resembles a combination of peak shaving and valley filling
Load levelling	Agamah and Ekonomou (2017a, b), Buja et al. (2017), Agamah and Ekonomou (2017a, b), Nikolaidis and Poullikkas (2018), David and Al-Anbagi (2017), Chin et al. (2017), Choe et al. (2017)	A strategy to transfer certain demands of one load to another usually based on a criticality factor	High-level achievement of system autonomy	Feasible only through flexible and critical load classifications	Exhibits characteristics of other DSM strategies
Energy arbitrage	Metz and Tomé (2018), Salles et al. (2016), Nikolaidis and Poullikkas (2018), Coronel et al. (2018), Cui et al. (2017), Yan et al. (2018), Wolfs et al. (2018), Touretzky and Baldea (2014), Carrion et al. (2018), Walawalkar et al. (2007), Berrueta et al. (2018), Alharbi and Bhattacharya (2018), Xie et al. (2019), Avilés et al. (2019), Molina (2017), Khalkhali and Hosseinian (2019)	Economic storage of cheaper energies for use or resale in periods of higher prices	Improve supply system reliability Reduce the rate of dump energies	Efficient energy storage management is needed In events of fully charged ESS, dump energies are likely to prevail	Very suitable for intermittent RE systems
Strategic conservation	Debnath et al. (2017), Eze et al. (2016), Gaur et al. (2017), Saini (2007), Romaní et al. (2018), Bastani et al. (2018), Khan (2019), Kumar and Harish (2014), Al-enezi (2010), Khan et al. (2016), Panapakidis et al. (2017a, b), Khalid et al. (n.d.)	Utility-based DR programme for customers to change usage patterns for incentives	A strategy for efficient use of energy	Customer tastes affect demand forecasts	Generally centred on reduced use of energy
Strategic load growth	Debnath et al. (2017), Eze et al. (2016), Attia (2010), Kumar (2014), Al-enezi (2010)	The planned increase in energy demands is due to the use of smart energy appliances	Minimise dump energies and energy cost savings	Only feasible in systems with dump loads It is never a standalone strategy, needs to be co-opted with other strategies such as valley filling	The strategy improves customer productivity while increasing utility sales
Flexible load scheduling	Tijjani et al. (2021)	A strategy with incentives and no definite shapes for deterioration of the system’s reliability	Good in improving the DG system’s autonomy	May not be feasible in systems of unified tariffs such as standalone	Most suitable in integrated systems with multi-tariff systems

**Table 6:** General concepts on tariff regimes discussed in the literature

Tariff regimes	References	Features	Merits	Demerits	Remarks
Standard residential tariff (SRT)	Ahluwalia and Bhatiani (2000), Linden et al. (2014)	■ Utility-based block form billings based on energy consumption, metre and equipment charges ■ Available in two structures applied to summer and winter seasons interchangeably	Generally good for energy consumption reduction	Billings is not specific to the energy consumed	The tariff structure is most suitable for vertically structured legacy grids
Conventional time-of-use (TOU)	Khan et al. (2016), Liu et al. (2019), Talent and Du (2018), Pan et al. (2019), Nikolaidis and Poullikkas (2018), Colmenar-Santos et al. (2017), Sharifi and Maghouli (2018), Reynolds et al. (2018), Chin et al. (2017), Chen et al. (2019)	A utility-initiated multi-block structure of electricity billings based on actual energy consumed	■ The billing system supports customer participation in the general development of the grid ■ The development aspect includes management of power generation, storage and retail	Mostly utility-centred Customers are subjected to higher costs of energy consumed	Major differences between conventional time-of-use (TOU) and standard residential tariffs (SRT) include the following ■ TOU has multi-block, while SRT has two block structures ■ SRT billings cover metre and equipment charges in addition to energy consumed
Real-time pricing (RTP)	Faria and Vale (2011), Fernandez et al. (2018), Panapakidis et al. (2017a, b), Zhu et al. (2019), Panapakidis et al. (2017a), Khan et al. 2016), Sharifi and Maghouli (2018), Erdinc et al. (2014), Rauf et al. (2020), Galisai et al. (2019), Guelpa et al. (2019)	■ A constantly changing electricity pricing mostly on an hourly basis ■ An attempt to improve conventional TOU structure by capturing the true cost of utility service	A true reflection of electricity billings that co-opts customer interests	■ Requires advanced metering and communication facilities ■ Block of customer swings likely to result in either overstretching demands or high dump energies	Generally viewed as a solution to TOU’s crude structure
Time-of-use Fitness		Considers energy billings based on real-time indices of demand criticalities and supply availability Options for customers’ use of energy based on real-time availability within the capacity of the RE autonomy or main grid imports Resembles RTP, but for RTPs customers’ billings are based on real-time generation initiated by the utility	Options for customers to defer non-critical loads to the regimes of RE system autonomy or periods of low energy costs	Conflicting techno-economic objectives between low cost of energy and RE system autonomy may prevail	An efficient method for customers to willingly use energy based on real-time criticality of demand

The factors of environment, economy and other social benefits continue to influence the proliferation of modern building system infrastructure as a global priority among most global societies. Hence, the importance of sustainability and energy conservation enhancement is emphasised among home users of energy. The sustainability and energy conservation emphasis is viewed here to influence electricity consumption patterns and affect customer comfort. It is worthy of note that the strategies used in energy management in buildings mainly focused on energy efficiency objectives. Hence, the BEMS is a strategic tool for intelligent grid management, as it allows interaction among building administrators, customers and utilities for monitoring, regulation and accountability in buildings’ energy utilisation (Sivaneasan et al. 2014).

The conventional time-of-use (${C}_{TOU}$) methods were considered in achieving load management schemes, as indicated by Pan et al. (2019), J. Liu and Zhong (2019), Rubaiee et al. (2019), Oprea et al. (2019) and Chen et al. (2019). Incidentally, the ${C}_{TOU}$ is fixed and does not usually appear to reflect the DSM-based desired operational objectives. It can be recalled that uncertain demand patterns hardly match RE resource distributions that periodically differ with the change in climatic conditions. This implies that the inflexibilities of ${C}_{TOU}$ may not be capable of enabling customer participation in optimal load management, where a close matching of stochastic RE generation and customer demands is the main goal. Methods such as real-time-pricing (RTP) can be considered in implementation of load management due to the customer-oriented features. Moreover, ${C}_{TOU}$ is utility-centred, and RTP emphasises on energy price per unit generation for a given time step. Hybridization of ${C}_{TOU}$ and RTP may enable customer’s full participation, through application of actual energy consumption charges. The proposed hybridization is envisaged to enable flexible options for customers’ decision on load scheduling. The benefits of load management expected to be achieved using the foregoing hybridization could be a multi-purpose strategy, with many DSM features such as the load shifting, peak shaving, energy arbitrage, strategic load growth and flexible load scheduling.

## Conclusions

In renewable energy (RE) systems, optimal planning and operation schemes are implemented using optimization techniques. The methods ensure economic selection and placement of the RE components based on the location’s resource availability. The RE system operations cover the implementation of power dispatch and demand side management (DSM) schemes. The power dispatch is strategic to RE system operations. However, the RE resource intermittence and demand uncertainties render power dispatch complex and uneconomical. Unstable climatic conditions also contribute to uncertainties in the RE resources and customer demands. An instance is the electricity demand for space comforts and lighting in buildings that seasonally vary. Hence, the DSM is preferred, as it follows supplies. The DSM also ensures customer participation in the system’s operational management. In emerging smart energy management systems such as the nearly zero energy buildings, the conflicting objectives of economy, environment and customer comforts are efficiently reconciled. Hence, the DSM implementations through demand response (DR) programmes concerning energy cost and utility tariffs are suggested using any of the suitable DSM strategies discussed. This implies that the DSM strategies can be implemented by integration with the customer-oriented tariffs for residential and commercial demands such as the conventional time-of-use (${C}_{TOU}$), real-time pricing (RTP) or both*.*

## Data Availability

The data used in the manuscripts is included in the text.

## References

[CR1] Abazari A, Monsef H, Wu B (2019). Coordination strategies of distributed energy resources including FESS, DEG, FC and WTG in load frequency control (LFC) scheme of hybrid isolated micro-grid. Int J Electr Power Energy Syst.

[CR2] Agamah SU & Ekonomou L (2017b) Peak demand shaving and load-levelling using a combination of bin packing and subset sum algorithms for electrical energy storage system scheduling. (March 2016). 10.1049/iet-smt.2015.0218

[CR3] Agamah SU, Ekonomou L (2017). Energy storage system scheduling for peak demand reduction using evolutionary combinatorial optimisation. Sustain Energy Technol Assess.

[CR4] Ahluwalia SS, Bhatiani G (2000) Tariff setting in the electric power sector base paper on Indian case study. Conference on Regulation in Infrastructure Services, pp 1–34. Available: https://www.semanticscholar.org/paper/Tariff-Setting-in-the-Electric-Power-Sector-Base-on-Ahluwalia-Bhatiani/504ebd84d1ff679071ad760bcfbb625e317649c2. Accessed 29 Jun 2019

[CR5] Akinyele D (2017). Techno-economic design and performance analysis of nanogrid systems for households in energy-poor villages. Sustain Cities Soc.

[CR6] Akmal M, El Kashif A, Ghazal M & Al Tarabsheh A. (2016). Demand response enabled sustainable smart home design in the middle east environment. EEEIC 2016 - International Conference on Environment and Electrical Engineering. 10.1109/EEEIC.2016.7555703

[CR7] Akram U, Khalid M, & Shafiq S (2018) An improved optimal sizing methodology for future autonomous residential smart power systems. IEEE Access 6. 10.1109/ACCESS.2018.2792451

[CR8] Al Dakheel J, Del Pero C, Aste N, Leonforte F (2020). Smart buildings features and key performance indicators a review. Sustain Cities Soc.

[CR9] Al Irsyad MI, Halog A, Nepal R (2019). Estimating the impacts of financing support policies towards photovoltaic market in Indonesia: A social-energy-economy-environment model simulation. J Environ Manage.

[CR10] Alekseeva N, Antoshkova N, Pupentsova S (2018). Application of the Monte Carlo simulation method in building and energy management systems. Energy management of municipal transportation facilities and transport.

[CR11] Al-enezi AN (2010) Demand side management (DSM) for efficient use of energy in the residential sector in Kuwait: analysis of options and priorities. De Montfort University, Leicester. http://hdl.handle.net/2086/4405. Accessed 29 Jun 2019

[CR12] Alharbi H, Bhattacharya K (2018). A goal programming approach to sizing and timing of third party investments in storage system for microgrids. 2018 IEEE Electrical Power and Energy Conference. EPEC.

[CR13] Amrr SM, Alam MS, Asghar MSJ, Ahmad F (2018). Low cost residential microgrid system based home to grid (H2G) back up power management. Sustain Cities Soc.

[CR14] Arasteh F, Riahy GH (2019). MPC-based approach for online demand side and storage system management in market based wind integrated power systems. Int J Electr Power Energy Syst.

[CR15] Atia R, Yamada N (2016). Sizing and analysis of renewable energy and battery systems in residential microgrids. IEEE Transactions on Smart Grid.

[CR16] Attia HA (2010). Mathematical formulation of the demand side management problem and its optimal solution. 14th International Middle East Power Systems Conference (MEPCON’10).

[CR17] Augusto C, Almeida RH, Mandelli S, Brito MC (2017). Evaluation of potential of demand side management strategies in isolated microgrid. 2017 6th International Conference on Clean Electrical Power: Renewable Energy Resources Impact. ICCEP.

[CR18] Avila F, Doris S, Valencia F (2015) Load modelling using affine arithmetic for demand side management. In: 2015 IEEE PES Innovative Smart Grid Technologies Latin America (ISGT LATAM), Montevideo, Uruguay, pp 456–460. 10.1109/ISGT-LA.2015.7381198

[CR19] Avilés AC, Oliva HS, Watts D (2019) Single-dwelling and community renewable microgrids: optimal sizing and energy management for new business models. Appl Energy 254:113665. 10.1016/j.apenergy.2019.113665

[CR20] Azim R, Cui H, Li F (2016). Power management strategy combining energy storage and demand response for microgrid emergency autonomous operation. Asia-Pacific Power and Energy Engineering Conference. APPEEC.

[CR21] Babar M, Tariq MU, Jan MA (2020). Secure and resilient demand side management engine using machine learning for IoT-enabled smart grid. Sustain Cities Soc.

[CR22] Baek J, Choi W, & Chae S (2017). Distributed control strategy for autonomous operation of hybrid AC/DC microgrid. Energies 10(3). 10.3390/en10030373

[CR23] Balakrishnan H, Tomar KKS, Singh SN (2017) An agent based approach for efficient energy management of microgrids. IEEE Region 10 Symposium (TENSYMP), Cochin, India, pp 1–5. 10.1109/TENCONSpring.2017.8070080

[CR24] Bastani M, Damgacioglu H, Celik N (2018). A δ-constraint multi-objective optimization framework for operation planning of smart grids. Sustain Cities Soc.

[CR25] Berardi U, Jafarpur P (2020) Assessing the impact of climate change on building heating and cooling energy demand in Canada. Renewable Sustainable Energy Rev 121:109681. 10.1016/j.rser.2019.109681

[CR26] Berrueta A, Heck M, Jantsch M, Ursúa A, Sanchis P (2018). Combined dynamic programming and region-elimination technique algorithm for optimal sizing and management of lithium-ion batteries for photovoltaic plants. Appl Energy.

[CR27] Bhatti AR, Salam Z (2018). A rule-based energy management scheme for uninterrupted electric vehicles charging at constant price using photovoltaic-grid system. Renew Energy.

[CR28] Bonilla D, Samaniego MG, Ramos R, Campbell H (2018). Practical and low-cost monitoring tool for building energy management systems using virtual instrumentation. Sustainable Cities and Society.

[CR29] Buja G, Bertoluzzo M, Fontana C (2017). Reactive power compensation capabilities of V2G-enabled electric vehicles. IEEE Trans Power Electron.

[CR30] Burmester D, Rayudu R, Seah W, Akinyele D (2017). A review of nanogrid topologies and technologies. Renew Sustain Energy Rev.

[CR31] Cabeza LF, Chàfer M (2020) Technological options and strategies towards zero energy buildings contributing to climate change mitigation: A systematic review. Energy Build 219:110009

[CR32] Calvillo CF, Sánchez-Miralles A, Villar J (2016). Energy management and planning in smart cities. Renew Sustain Energy Rev.

[CR33] Carrion M, Dvorkin Y, Pandzic H (2018). Primary frequency response in capacity expansion with energy storage. IEEE Trans Power Syst.

[CR34] Cetin KS, Novoselac A (2015). Single and multi-family residential central all-air HVAC system operational characteristics in cooling-dominated climate. Energy Build.

[CR35] Chen SH, Liou YC, Chen YH, Wang KC (2019). Order acceptance and scheduling problem with carbon emission reduction and electricity tariffs on a single machine. Sustainability.

[CR36] Chin JX, Tinoco De Rubira T, Hug G (2017). Privacy-protecting energy management unit through model-distribution predictive control. IEEE Trans Smart Grid.

[CR37] Choe J, Choe G & Lai J (2017) System for load levelling control and operation of an energy storage system. 739–745. 10.1049/iet-pel.2016.0458

[CR38] Colmenar-Santos A, de Palacio-Rodriguez C, Rosales-Asensio E, Borge-Diez D (2017). Estimating the benefits of vehicle-to-home in islands: the case of the Canary Islands. Energy.

[CR39] Cordova-Fajardo MA & Tututi ES (2019). Incorporating home appliances into a DC home nanogrid. J Physics: Conf Series 1221(1). 10.1088/1742-6596/1221/1/012048

[CR40] Coronel T, Buzarquis E, Blanco GA (2018) Analyzing feasibility of energy storage system for energy arbitrage. In: CHILEAN Conference on Electrical, Electronics Engineering. Information and Communication Technologies (CHILECON), Pucon, Chile, pp 1–6. 10.1109/CHILECON.2017.8229547

[CR41] Craparo EM, Sprague JG (2019). Integrated supply- and demand-side energy management for expeditionary environmental control. Appl Energy.

[CR42] Cui H, Li F, Fang X, Chen H, Wang H (2017). Bi-level arbitrage potential evaluation for grid-scale energy storage considering wind power and lmp smoothing effect. IEEE Trans Sustain Energy.

[CR43] Dahiru AT (2021) Nanogrid sizing using nested integer linear programming and time-of-use based load management. Universiti Teknologi Malaysia

[CR44] David AO, Al-Anbagi I (2017). EVs for frequency regulation: cost benefit analysis in a smart grid environment. IET Electr Syst Transp.

[CR45] Debnath R, Kumar D & Mohanta DK (2017). Effective demand side management (DSM) strategies for the deregulated market envioronments. 2017 Conference on Emerging Devices and Smart Systems, ICEDSS 2017 March 110–115. 10.1109/ICEDSS.2017.8073668

[CR46] Deckmyn C, Van de Vyver J, Vandoorn TL, Meersman B, Desmet J, Vandevelde L (2017). Day-ahead unit commitment model for microgrids. IET Gener Transm Distrib.

[CR47] Degha HE, Laallam FZ, Said B (2019). Intelligent context-awareness system for energy efficiency in smart building based on ontology. Sustain Comput: Inform Syst.

[CR48] Erdinc O, Paterakis N, Catalao JPS, Bakirtzis AG (2014) An ANFIS based assessment of demand response driven load pattern elasticity. IEEE Power and Energy Society General Meeting. 10.1109/PESGM.2014.6939324

[CR49] Eze C, Agwu D, Uzoechi LO (2016). A new proposed demand side management technique. Int J Eng Sci Emerg Technol.

[CR50] Faria P, Vale Z (2011). Demand response in electrical energy supply: an optimal real time pricing approach. Energy.

[CR51] Farrokhifar M, Bahmani H, Faridpak B, Safari A, Pozo D, Aiello M (2021). Model predictive control for demand side management in buildings a survey. Sustain Cities Soc.

[CR52] Fernandez E, Hossain MJ, Nizami MSH (2018). Game-theoretic approach to demand-side energy management for a smart neighbourhood in Sydney incorporating renewable resources. Appl Energy.

[CR53] Flores-Larsen S, Filippín C, Barea G (2019). Impact of climate change on energy use and bioclimatic design of residential buildings in the 21st century in Argentina. Energy Build.

[CR54] Friedman JP, Harris JC, Lindeman JB (2017) Dictionary of real estate terms. Simon and Schuster, London. Available: https://books.google.com.ng/books?id=WkxKNIx7HQoC&dq=dictionary+of+real+estate+terms&hl=en&sa=X&ved=2ahUKEwjysuXN0cT8AhVXTaQEHZsWC0EQ6AF6BAgIEAI. Accessed 22 Dec 2022

[CR55] Galisai S, Ghiani E, Pilo F (2019) Multi-objective and multi-criteria optimization of microgrids for nearly zero-energy buildings. SEST 2019 - 2nd International Conference on Smart Energy Systems and Technologies 1:1–6. 10.1109/SEST.2019.8849103

[CR56] Ganesan S, Padmanaban S, Varadarajan R, Subramaniam U & Mihet-Popa L (2017). Study and analysis of an intelligent microgrid energy management solution with distributed energy sources. Energies 10(9). 10.3390/en10091419

[CR57] Gaur G, Mehta N, Khanna R, Kaur S (2017) Demand side management in a smart grid environment. IEEE International Conference on Smart Grid and Smart Cities (ICSGSC), Singapore, pp 227-231. 10.1109/ICSGSC.2017.8038581

[CR58] Genikomsakis K, Lopez S, Dallas P, Ioakimidis C (2017). Simulation of wind-battery microgrid based on short-term wind power forecasting. Appl Sci.

[CR59] Gernaat DEHJ, de Boer HS, Daioglou V (2021). Author Correction: Climate change impacts on renewable energy supply. Nat Clim Chang.

[CR60] Guelpa E, Marincioni L, Cheng P, Huang T, Chien Y, Wu C, Tai C, Fu L, Zhu H, Gao Y, Hou Y, Wang Z, Feng X, Su H, Zio E, Zhang J, Chi L, Li X, Zhang Z (2019). A systematic data-driven demand side management method for smart natural gas supply systems. Electr Power Energy Syst.

[CR61] Hoogsteen G, Van Der Klauw T, Molderink A, Hurink JL, Smit GJM, Feng X & Hebner RE (2016) Balancing islanded residential microgrids using demand side management. 2016 IEEE Power and Energy Society Innovative Smart Grid Technologies Conference, ISGT 2016. 10.1109/ISGT.2016.7781167

[CR62] Hossain MA, Pota HR, Hossain MJ, Haruni AMO (2018). Active power management in a low-voltage islanded microgrid. Int J Electr Power Energy Syst.

[CR63] Hu S, Yan D, Azar E, Guo F (2020). A systematic review of occupant behavior in building energy policy. Build Environ.

[CR64] Insam E (2017). Optimal sizing of stand-alone renewable energy systems for electricity & fresh water supply.

[CR65] Ioakimidis CS, Thomas D, Rycerski P, Genikomsakis KN (2018). Peak shaving and valley filling of power consumption profile in non-residential buildings using an electric vehicle parking lot. Energy.

[CR66] IRENA (2018) Policies and regulations for renewable mini-grids. Available: https://irena.org/-/media/Files/IRENA/Agency/Publication/2018/Oct/IRENA_mini-grid_policies_2018.pdf. Accessed 30 Jan 2019

[CR67] Islam FR, Prakash K, Mamun KA, Lallu A, & Pota HR (2017). Aromatic network: a novel structure for power distribution system. IEEE Access 5. 10.1109/ACCESS.2017.2767037

[CR68] Jabir H, Teh J, Ishak D, Abunima H (2018). Impacts of demand-side management on electrical power systems: a review. Energies.

[CR69] Jacob AS, Banerjee R, Ghosh PC (2018). Sizing of hybrid energy storage system for a PV based microgrid through design space approach. Appl Energy.

[CR70] Jagun ZT (2020). Risks in feasibility and viability appraisal process for property development and the investment market in Nigeria. J Prop Invest Finance.

[CR71] Javaid N, Hafeez G, Iqbal S Alrajeh N, Alabed MS & Guizani M (2018) Energy efficient integration of renewable energy sources in the smart grid for demand side management. IEEE Access, PP(c):1. 10.1109/ACCESS.2018.2866461

[CR72] Khalid A, Javaid N, Guizani M, Alhussein M, Aurangzeb K & Ilahi M (n.d.). Towards dynamic coordination among home appliances using multi-objective energy optimization for demand side management in smart buildings. IEEE Access 1–1. 10.1109/ACCESS.2018.2791546

[CR73] Khalkhali H, Hosseinian SH (2019). Novel residential energy demand management framework based on clustering approach in energy and performance-based regulation service markets. Sustainable Cities and Society.

[CR74] Khan AR, Mahmood A, Safdar A, Khan ZA, Khan NA (2016). Load forecasting, dynamic pricing and DSM in smart grid: a review. Renew Sustain Energy Rev.

[CR75] Khan I (2019). Energy‑saving behaviour as a demand‑side management strategy in the developing world: the case of Bangladesh. International J Energy Environ Eng 0123456789. 10.1007/s40095-019-0302-3

[CR76] Kou P, Liang D, Gao L (2018). Stochastic energy scheduling in microgrids considering the uncertainties in both supply and demand. IEEE Syst J.

[CR77] Kuang Y, Zhang Y, Zhou B, Li C, Cao Y, Li L, Zeng L (2016). A review of renewable energy utilization in islands. Renew Sustain Energy Rev.

[CR78] Kumar J, Suryakiran BV, Verma A, Bhatti TS (2019). Analysis of techno-economic viability with demand response strategy of a grid-connected microgrid model for enhanced rural electrification in Uttar Pradesh state, India. Energy.

[CR79] Kumar A, Harish VSKV (2014) Planning and implementation strategy of Demand Side Management in India. First International Conference on Automation, Control, Energy and Systems (ACES), Adisaptagram, India, pp 1–6. 10.1109/ACES.2014.6808001

[CR80] Kylili A, Fokaides PA (2020). European smart cities : the role of zero energy buildings. Sustain Cities Soc.

[CR81] Li B, Roche R, Paire D, Miraoui A (2017). Sizing of a stand-alone microgrid considering electric power, cooling/heating, hydrogen loads and hydrogen storage degradation. Appl Energy.

[CR82] Li W, Zhang G, Ai L, Liu G, Gao Z, Liu H (2019). Characteristics analysis at high speed of asynchronous axial magnetic coupler for superconducting flywheel energy storage system. IEEE Trans Appl Supercond.

[CR83] Linden AJ, Kalantzis F, Maincent E, Pienkowski J (2014) Electricity tariff deficit: temporary or permanent problem in the EU? In: European Commission Economic Papers, vol. 534. Directorate General for Economic and Financial Affairs, European Commission. 10.2765/71426

[CR84] Liu M, Phanivong PK, Shi Y & Callaway DS (2017). Decentralized charging control of electric vehicles in residential distribution networks. IEEE Trans Control Syst Technol 1–16. 10.1109/TCST.2017.2771307

[CR85] Liu P, Cai Z, Xie P, Li X & Zhang Y (2019). A computationally effcient optimization method for battery storage in grid-connected microgrids based on a power exchanging process. Energies 12(8). 10.3390/en12081512

[CR86] Liu J, Zhong C (2019). An economic evaluation of the coordination between electric vehicle storage and distributed renewable energy. Energy.

[CR87] Lokeshgupta B, Sivasubramani S (2018) Multi-objective dynamic economic and emission dispatch with demand side management. Int J Electr Power Energy Syst 97:334–343. 10.1016/j.ijepes.2017.11.020

[CR88] Loucks DP, van Beek E (2017) Water resource systems planning and management: an introduction to methods, models, and applications. In: Water resource systems planning and management: an introduction to methods, models, and applications. 10.1007/978-3-319-44234-1

[CR89] Ma Y, Li B (2020) Hybridized intelligent home renewable energy management system for smart grids. In Sustainability (Switzerland) 12(5). 10.3390/su12052117

[CR90] Ma K, Hu S, Yang J, Dou C & Guerrero JM (2017). Energy trading and pricing in microgrids with uncertain energy supply: a three-stage hierarchical game approach. Energies 10(5). 10.3390/en10050670

[CR91] Majidi M & Zare K (2018) Integration of smart energy hubs in distribution networks under uncertainties and demand response concept. IEEE Trans Power Syst PP(c):1. 10.1109/TPWRS.2018.2867648

[CR92] Mallol-Poyato R, Jiménez-Fernández S, Díaz-Villar P, Salcedo-Sanz S (2016). Joint optimization of a microgrid’s structure design and its operation using a two-steps evolutionary algorithm. Energy.

[CR93] Mariano-Hernández D, Hernández-Callejo L, Zorita-Lamadrid A, Duque-Pérez O & Santos García F (2021) A review of strategies for building energy management system: model predictive control, demand side management, optimization, and fault detect & diagnosis. J Build Eng 33(July 2020). 10.1016/j.jobe.2020.101692

[CR94] Märkle-Huß J, Feuerriegel S, Neumann D (2018). Large-scale demand response and its implications for spot prices, load and policies: insights from the German-Austrian electricity market. Appl Energy.

[CR95] Martirano L, Habib E, Parise G, Greco G, Manganelli M, Massarella F, Parise L (2017). Demand side management in microgrids for load control in nearly zero energy buildings. IEEE Trans Ind Appl.

[CR96] Masters GM (2004) Renewable and efficient electric power systems. In: IEEE Press (ed.) 2nd ed. John Wiley & Sons, Inc. 10.1002/0471668826

[CR97] Mendes PRC, Maestre M, Bordons C, Normey-rico JE (2016) Binary Search Algorithm for mixed integer optimization: application to energy management in a microgrid. In: 2016 European Control Conference (ECC), Aalborg, Denmark, pp 2620–2625. 10.1109/ECC.2016.7810685

[CR98] Metz D, Tomé J (2018). Use of battery storage systems for price arbitrage operations in the 15- and 60-min German intraday markets. Electric Power Syst Res.

[CR99] Molderink A, Bakker V, Bosman MGC, Hurink JL, Smit GJM (2010). Management and control of domestic smart grid technology. IEEE Trans Smart Grid.

[CR100] Molderink A, Member S, Bakker V, Bosman MGC, Hurink JL, Smit GJM (2010). Manag Control Domest Smart Grid Technol.

[CR101] Molina MG (2017). Energy storage and power electronics technologies: a strong combination to empower the transformation to the smart grid. Proc IEEE.

[CR102] Momoh J (2012) Smart grid: fundamentals of design and analysis. In: IEEE Press, Publication. IEEE Press A John Wiley & Sons, Inc.

[CR103] Monyei CG & Adewumi AO (n.d.) Integration of demand side and supply side energy management resources for optimal scheduling of demand response loads – South Africa in focus. Electr Power Syst Res 158, 92–104. 10.1016/j.epsr.2017.12.033

[CR104] Mortaz E, Vinel A, Dvorkin Y (2019). An optimization model for siting and sizing of vehicle-to-grid facilities in a microgrid. Appl Energy.

[CR105] Moussa S, Ghorbal MJB, Slama-Belkhodja I (2019). Bus voltage level choice for standalone residential DC nanogrid. Sustain Cities Soc.

[CR106] Nazarloo A, Feyzi MR, Sabahi M & Bannae MB (2018). Improving voltage profile and optimal scheduling of vehicle to grid energy based on a new method. 18(1):81–88

[CR107] Nikolaidis P, Poullikkas A (2018). Cost metrics of electrical energy storage technologies in potential power system operations. Sustain Energy Technol Assess.

[CR108] Nordman B (2009) Nanogrids: evolving our electricity systems from the bottom up. In Darnell Green Power Forum. Available: https://www.semanticscholar.org/paper/Nanogrids%3A-Evolving-our-electricity-systems-from-up-Nordman-Lawrence/9886328689c0261ec42d2b73ea9dcdfb90ac6ec8. Accessed 29 Apr 2020

[CR109] Nunna HSVSK, Doolla S (2013). Intelligent demand side management in smart-microgrids. Proceedings - 2013 IEEE International Workshop on Intelligent Energy Systems. IWIES.

[CR110] Oh HS (2018) Demand-side management with a state space consideration. Energies 11(9). 10.3390/en11092444

[CR111] Oprea SV, Bâra A, Ifrim GA, Coroianu L (2019). Computers & industrial engineering day-ahead electricity consumption optimization algorithms for smart homes. Comput Ind Eng.

[CR112] Pan R, Li Z, Cao J, Zhang H, Xia X (2019). Computers & Industrial engineering electrical load tracking scheduling of steel plants under time-of-use tariffs. Comput Ind Eng.

[CR113] Panapakidis IP, Christoforidis GC, Asimopoulos N & Dagoumas AS (2017a). A novel demand side management strategy implementation utilizing real-time pricing schemes. Conference Proceedings - 2017a 17th IEEE International Conference on Environment and Electrical Engineering and 2017a 1st IEEE Industrial and Commercial Power Systems Europe, EEEIC / I and CPS Europe 2017. 10.1109/EEEIC.2017.7977758

[CR114] Panapakidis IP, Christoforidis GC, Asimopoulos N & Dagoumas AS (2017b) A novel demand side management strategy implementation utilizing real-time pricing schemes. Conference Proceedings - 2017b 17th IEEE International Conference on Environment and Electrical Engineering and 2017b 1st IEEE Industrial and Commercial Power Systems Europe, EEEIC / I and CPS Europe 2017. 10.1109/EEEIC.2017.7977758

[CR115] Pannala S, Padhy N, Agarwal P (2017). Peak energy management using renewable integrated DC microgrid. IEEE Trans Smart Grid.

[CR116] Tudu B, Mandal KK, Chakraborty N (2019). Optimal design and development of PV-wind-battery based nano-grid system: A field-on-laboratory demonstration. Front Energy.

[CR117] Program JUBESMA, ESMAP (2000) Mini-grid design manual. Available: https://documents1.worldbank.org/curated/en/730361468739284428/pdf/multi-page.pdf. Accessed 29 Jun 2019

[CR118] Rauf S, Kalair AR, Khan N (2020). Variable load demand scheme for hybrid AC/DC nanogrid. Int J Photoenergy.

[CR119] Reynolds J, Rezgui Y, Kwan A, Piriou S (2018). A zone-level, building energy optimisation combining an artificial neural network, a genetic algorithm, and model predictive control. Energy.

[CR120] Romaní J, Belusko M, Alemu A, Cabeza LF, de Gracia A, Bruno F (2018). Control concepts of a radiant wall working as thermal energy storage for peak load shifting of a heat pump coupled to a PV array. Renew Energy.

[CR121] Rubaiee S, Cinar S, Yildirim MB (2019). An energy-aware multiobjective optimization framework to minimize total tardiness and energy cost on a single-machine nonpreemptive scheduling. IEEE Trans Eng Manage.

[CR122] Saini S (2007) Demand-side management module. In Sustainable Energy Regulation and Policymaking for Africa. Available: https://www.unido.org/sites/default/files/2009-02/Module14_0.pdf. Accessed 29 Jun 2019

[CR123] Salles MBC, Aziz MJ, Hogan WW (2016) Potential arbitrage revenue of energy storage systems in PJM during 2014. In: IEEE Power and Energy Society General Meeting. Available: 10.1109/PESGM.2016.7741114

[CR124] Sellali M, Betka A, Abdedaim S, Ouchen S (2017) Implementation of a real-time energy management consisting of a battery and a supercapacitor. In: 2017 5th International Conference on Electrical Engineering - Boumerdes (ICEE-B), Boumerdes, Algeria, pp 1–6. 10.1109/ICEE-B.2017.8192200

[CR125] Sepulveda C, Canha L, Sperandio Mauricio S, R.  (2018). Methodology for ESS-type selection and optimal energy management in distribution system with DG considering reverse flow limitations and cost penalties. IET Gen Transm Distrib.

[CR126] Serpi A, Porru M, & Damiano A (2017). An optimal power and energy management by hybrid energy storage systems in microgrids. Energies, 10(11). 10.3390/en10111909

[CR127] Shahidehpour M, Li Z, Gong W, Bahramirad S, Lopata M (2017). A Hybrid ac\/dc nanogrid: the keating hall installation at the Illinois Institute of Technology. IEEE Electrification Mag.

[CR128] Sharifi AH, Maghouli P (2018). Energy management of smart homes equipped with energy storage systems considering the PAR index based on real-time pricing. Sustain Cities Soc.

[CR129] Sivaneasan B, Kumar K, Nandha, Tan KT, So PL (2014). Preemptive demand response management for buildings. IEEE Trans Sustain Energy. 10.1109/TSTE.2014.2375895

[CR130] Solaun K, Cerdá E (2019) Climate change impacts on renewable energy generation. A review of quantitative projections. Renewable Sustainable Energy Rev 116:109415. 10.1016/j.rser.2019.109415

[CR131] SoltaniNejad Farsangi A, Hadayeghparast S, Mehdinejad M, Shayanfar H (2018). A novel stochastic energy management of a microgrid with various types of distributed energy resources in presence of demand response programs. Energy.

[CR132] Strnad I, Prenc R (2017). Optimal sizing of renewable sources and energy storage in low-carbon microgrid nodes. Electr Eng.

[CR133] Sun B, Huang Z, Tan X, Tsang DHK (2016). Optimal scheduling for electric vehicle charging with discrete charging levels in distribution grid. IEEE Trans Smart Grid.

[CR134] Talent O, Du H (2018). Optimal sizing and energy scheduling of photovoltaic-battery systems under different tariff structures. Renew Energy.

[CR135] Thomsen J (2018). Enhancing operation of decentralized energy systems by a regional economic optimization model DISTRICT. In Energy Systems (Vol. 9, Issue 3). Springer Berlin Heidelberg. 10.1007/s12667-017-0261-9

[CR136] Tijjani A, Wei C, Lawan A, Yiew K (2021). Energy cost reduction in residential nanogrid under constraints of renewable energy, customer demand fitness and binary battery operations. J Energy Storage.

[CR137] Touretzky CR, Baldea M (2014). With energy storage. J Process Control.

[CR138] Tu T, Rajarathnam GP, Vassallo AM (2019). Optimization of a stand-alone photovoltaic–wind–diesel–battery system with multi-layered demand scheduling. Renew Energy.

[CR139] Uddin M, Romlie MF, Abdullah MF, Abd Halim S, Abu Bakar AH, Chia Kwang T (2018). A review on peak load shaving strategies. Renew Sustain Energy Rev.

[CR140] Urbanucci L (2018). Limits and potentials of mixed integer linear programming methods for optimization of polygeneration energy systems. Energy Procedia.

[CR141] Walawalkar R, Apt J, Mancini R (2007). Economics of electric energy storage for energy arbitrage and regulation in New York. Energy Policy.

[CR142] Wang J, Tang G, Huang JX (2018). Analysis and modelling of a novel hydrostatic energy conversion system for seabed cone penetration test rig. Ocean Eng.

[CR143] Wang Y, Li Y, Cao Y, Tan Y, He L, Han J (2018). Hybrid AC/DC microgrid architecture with comprehensive control strategy for energy management of smart building. International Journal of Electrical Power and Energy Systems.

[CR144] Wittenberg I, Matthies E (2018). How do PV households use their PV system and how is this related to their energy use?. Renew Energy.

[CR145] Wolfs P, Emami K, Lin Y, Palmer E (2018). Load forecasting for diurnal management of community battery systems. J Modern Power Syst Clean Energy.

[CR146] Xie H, Teng X, Xu Y, Wang Y (2019). Optimal energy storage sizing for networked microgrids considering reliability and resilience. IEEE Access.

[CR147] Yaghmaee MH, Moghaddassian M, Leon-Garcia A (2017). Autonomous two-tier cloud-based demand side management approach with microgrid. IEEE Trans Industr Inf.

[CR148] Yan X, Gu C, Wyman-Pain H, Li F (2018). Optimal capacity management for multi-service energy storage in market participation using portfolio theory. IEEE Transactions on Industrial Electronics.

[CR149] Yang X, Zhang Y, Wu H & He H (2018). An event-driven ADR approach for residential energy resources in microgrids with uncertainties. IEEE Trans Ind Electr PP(APRIL):1. 10.1109/TIE.2018.2868019

[CR150] Yang XS, He X (2016) Nature-inspired optimization algorithms in engineering: overview and applications. Nature-inspired Computation in Engineering, pp 1–20

[CR151] Zenginis I, Vardakas JS, Echave C, Morató M, Abadal J, Verikoukis CV (2017). Cooperation in microgrids through power exchange: an optimal sizing and operation approach. Appl Energy.

[CR152] Zhang F, Zhao H, Hong M (2015). Operation of networked microgrids in a distribution system. CSEE J Power Energy Syst.

[CR153] Zhang Q, Deng W, Li G (2017). Stochastic control of predictive power management for battery/supercapacitor hybrid energy storage systems of electric vehicles. IEEE Trans Ind Inform.

[CR154] Zhu H, Gao Y, Hou Y, Wang Z, Feng X (2019). Electrical power and energy systems real-time pricing considering different type of smart home appliances based on Markov decision process. Electr Power Energy Syst.

[CR155] Zhai ZJ, Helman JM (2019). Implications of climate changes to building energy and design. Sustainable Cities and Society.

